# Community size structure varies with predator–prey size relationships and temperature across Australian reefs

**DOI:** 10.1002/ece3.8789

**Published:** 2022-04-07

**Authors:** Amy Rose Coghlan, Julia L. Blanchard, Freddie J. Heather, Rick D. Stuart‐Smith, Graham J. Edgar, Asta Audzijonyte

**Affiliations:** ^1^ 3925 Institute for Marine and Antarctic Studies (IMAS) University of Tasmania Hobart Tasmania Australia; ^2^ 3925 Centre for Marine Socioecology University of Tasmania Hobart Tasmania Australia

**Keywords:** coastal ecosystems, community composition, habitat complexity, predation, predator–prey mass ratio, size spectrum

## Abstract

Climate change and fisheries exploitation are dramatically changing the abundances, species composition, and size spectra of fish communities. We explore whether variation in ‘abundance size spectra’, a widely studied ecosystem feature, is influenced by a parameter theorized to govern the shape of size‐structured ecosystems—the relationship between the sizes of predators and their prey (predator–prey mass ratios, or PPMRs). PPMR estimates are lacking for avast number of fish species, including at the scale of trophic guilds. Using measurements of 8128 prey items in gut contents of 97 reef fish species, we established predator–prey mass ratios (PPMRs) for four major trophic guilds (piscivores, invertivores, planktivores, and herbivores) using linear mixed effects models. To assess the theoretical predictions that higher community‐level PPMRs leads to shallower size spectrum slopes, we compared observations of both ecosystem metrics for ~15,000 coastal reef sites distributed around Australia. PPMRs of individual fishes were remarkably high (median ~71,000), with significant variation between different trophic guilds (~890 for piscivores; ~83,000 for planktivores), and ~8700 for whole communities. Community‐level PPMRs were positively related to size spectrum slopes, broadly consistent with theory, however, this pattern was also influenced by the latitudinal temperature gradient. Tropical reefs showed a stronger relationship between community‐level PPMRs and community size spectrum slopes than temperate reefs. The extent that these patterns apply outside Australia and consequences for community structure and dynamics are key areas for future investigation.

## Graphical abstract

We explore whether variation in abundance size spectra, a widely established community metric in ecology, is influenced by the relationship between the sizes of predators and their prey. Using measurements of 8128 prey items in gut contents of 97 reef fish species, we established predator–prey mass ratios (PPMRs) for four major trophic guilds (piscivores, invertivores, planktivores, and herbivores) using linear mixed effects models. To assess theoretical predictions that higher mean community‐level PPMR leads to shallower size spectrum slopes, we compared observations of mean community‐level PPMR with size spectrum slopes for coastal reef sites distributed around Australia.

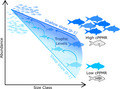

## 1 INTRODUCTION

Despite accounting for only ~6% of the global surface, coastal seas contribute ~40% of estimated global ecosystem services (Costanza et al., 1998) and support commercial, recreational, and artisanal fisheries worldwide. Currently, fisheries and climate change are causing dramatic changes in the species composition and body size structure of coastal fish communities (Audzijonyte et al., 2016, 2020; Waples & Audzijonyte, 2016). Given body size is the single‐most important biological trait determining both an organism's vital rates (metabolism, respiration, and development) and ecological interactions (movement capacity, predation risk, and trophic position) (Peters, 1983), changes in the size structure of predators and prey can have major implications for ecosystem functioning. This is particularly so for marine ecosystems as many marine animals increase in body size by several orders of magnitude from larva to adult (Sibly et al., 2015), thus the subsequent ecological interactions of these individuals are highly subject to changes in predator and prey size structure (Sánchez‐Hernández et al., 2019, and references therein).

Ecological community size structure is often described through ‘size spectra’, where the number of individuals (or their summed biomass) is shown in relation to body size classes (Sheldon et al., 1972; Sprules & Barth, 2016). In the absence of fishing, both empirical and theoretical studies have shown that abundance declines with body size with a slope close to −1, corresponding to roughly equal biomass in size class bins on a logarithmic scale (Blanchard et al., 2017; Sprules & Barth, 2016). However, despite its conservative nature, several factors can affect size spectra, most notably the selective removal of larger‐bodied individuals (e.g., via fishing), which results in fewer larger‐bodied individuals relative to smaller‐bodied individuals, thus steeper size spectrum slopes (Dulvy et al., 2004; Graham et al., 2005; Robinson et al., 2017); along with increasing temperature (Blanchard et al., 2005; Pomeranz et al., 2022) and pollution (Arranz et al., 2021). The slope of size spectra provides a useful indicator of reef ecosystem health, and an improved understanding of ecological size spectra baselines and responses to different pressures is needed (Nash & Graham, 2016).

A critical parameter governing theoretical community size spectra is the ratio between a predator's body size or mass and that of its prey (‘Predator Prey Mass Ratio’, PPMR) (Andersen, 2019; Andersen, Berge, et al., 2016; Andersen Jacobsen et al., 2016; Jennings et al., 2002). Higher PPMR values (>1) indicate that a predator is consuming prey relatively smaller than itself, whereas lower PPMRs (closer to 1) indicate that a predator consumes prey closer to its own body size (and often, trophic level) (Jennings & Mackinson, 2003; Jennings et al., 2007; Jennings & Warr, 2003). PPMRs <1, meanwhile, suggest a predator is consuming prey larger than itself, a predation strategy (including work‐around such as ‘pack hunting’ to take down larger prey) which is remarkably uncommon in marine systems compared to size‐constrained predation (Trebilco et al., 2013; Woodson et al., 2018). The upper limit of prey size in many marine predators is set by ‘gape‐limitation’, where predators are restricted to only consuming prey that can fit through their jaws whole (Mihalitsis & Bellwood, 2017). Below this upper size limit, many gape‐limited predators feed on a wide range of prey sizes, depending on predator traits such as morphology, behavior, and body size (Barnes et al., 2010; Scharf et al., 2000), which leads to considerable variation in individual‐level PPMRs. By averaging PPMR values across the range of individuals (and traits) that compose a community (deriving community level or cPPMR), insights can be gained into the energetics and functioning of the broader system (Bellwood et al., 2020; Dornburg et al., 2017; Troudet et al., 2017), including the number of trophic levels possible in the food web, and the steepness of size spectrum slopes.

According to the ‘Energetic Equivalence Hypothesis with Trophic Transfer Correction’, the unexploited biomass size spectrum slope (*b*) and abundance size spectrum slope (*b*−1) can be estimated with just two key community parameters—the cPPMR and trophic efficiency (*TE)* (Jennings & Mackinson, 2003; Reuman et al., 2009):

(1) $$b=0.25+\;\frac{\log(TE)}{\log(cPPMR)}$$

Here, ‘0.25’ accounts for the average scaling of an animal's metabolic rate as body mass increases (Brown et al., 2004; Sibly et al., 2015; Von Bertalanffy, 1957). *TE* describes the average proportion of biomass transferred between trophic levels, and *cPPMR* is the community‐averaged individual PPMR values (equation 1). In this equation, TE and cPPMR are considered independent, that is, energy transfer efficiency is not influenced by cPPMR values (Reuman et al., 2009). Several constraints apply to the magnitude of the values in equation (1) and consequently restrict the possible range of ‘*b*’ (Trebilco et al., 2013; Woodson et al., 2018). TE, for instance, cannot exceed 1 (i.e., due to the laws of thermodynamics, a predator cannot gain more energy than is present within the prey) and is often considered to be ~0.1 due to energy losses in the capture, handling and digestion of prey, along with the metabolic costs of the predator (Andersen et al., 2009; Lindeman, 1942 although see Eddy et al., 2021).

**FIGURE 1:**
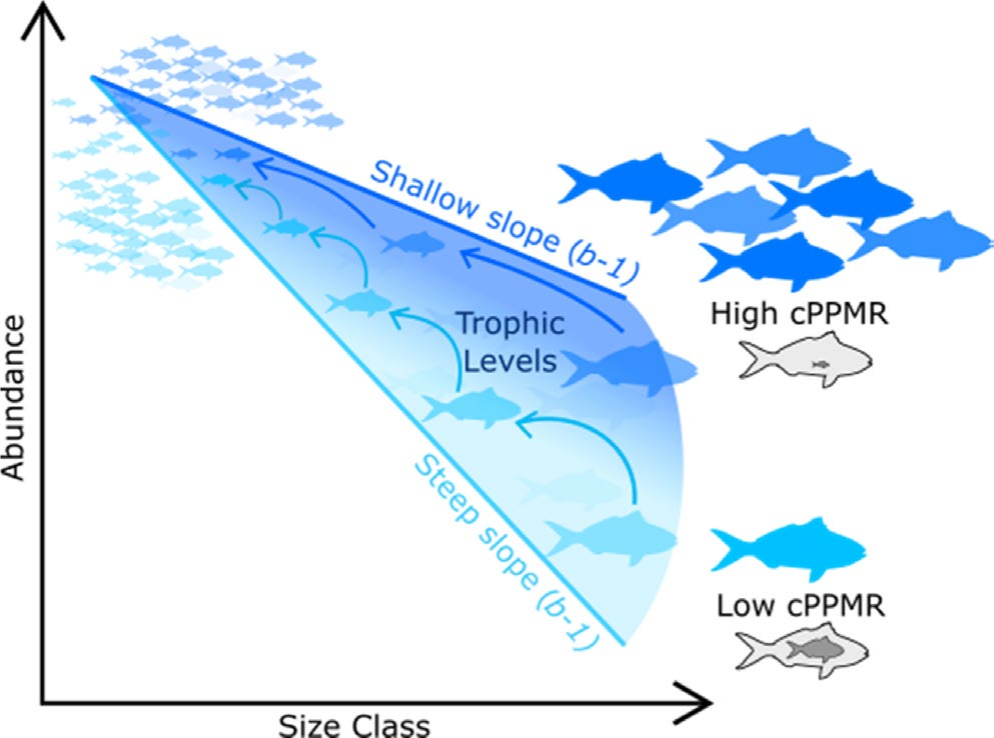
Conceptual diagram illustrating relationship between community size spectrum slope and cPPMR (assuming the same transfer efficiency (TE) across trophic levels, and similar abundances at the smallest size class). The abundance of large‐bodied fish is greater when cPPMR is high (represented by the relatively small size of prey in the outlined fish), with large fish abundance decreasing with lower cPPMR, resulting in steep, or shallow, abundance size spectrum slopes (*b*−1). High cPPMR is further associated with fewer steps in the food chain as large‐bodied fish consume relatively smaller prey, leaving fewer trophic steps in between, and fewer trophic levels overall

While cPPMR is generally >1 for marine fishes, empirical estimates of cPPMR rely on dietary or stable isotope data which are inherently difficult to attain for whole communities, and consequently such information is widely unavailable. The few empirical cPPMR compilations that do exist for marine environments range from 390 for the North Sea shelf (Jennings & Blanchard, 2004), to 1047 for a Bahamian tropical reef (Zhu et al., 2019), 1650 for a kelp forest reef (Trebilco et al., 2016), and 7792 for a tropical reef in the Western Arabian Sea (Al‐Habsi et al., 2008). Importantly, across a cPPMR range of 100–10,000, when TE is 0.1, equation (1) predicts biomass size spectrum slopes ‘*b*’ that span both negative and positive values (Trebilco et al., 2016), the latter resulting in ‘top‐heavy’ ecosystems and relatively higher abundance of larger‐ to smaller‐sized individuals (Jennings & Mackinson, 2003; Trebilco et al., 2013; Woodson et al., 2018), often cited for ‘pristine’ marine ecosystems (McCauley et al., 2018; Trebilco et al., 2013, 2016; Woodson et al., 2018). Many reefs are dominated by herbivores, invertivores, planktivores, and detritivores, that may consume small‐bodied prey throughout their lifetime (resulting in higher PPMRs when they become larger). The consumption of relatively small prey may energetically permit these trophic guilds to become more abundant at large sizes (Woodson et al., 2018), leading to shallower community size spectrum slopes (Figure 1).

The contribution of differences in cPPMR to variation in abundance size spectrum slopes has not been empirically assessed across large scales. Globally, empirical abundance size spectra have generally been found to approach the expected slopes (*b*−1) of approximately −1 (Heather et al., 2021). This applies to lightly exploited reef communities when including both fishes and invertebrates. Yet, there was also reasonable variation in slope estimates across reef sites and locations (min: −2.5; max: 2.1 Heather, Blanchard, et al., 2021). Such variation may result from large differences in the community composition at sites, such that the relative contribution of fish groups with low (e.g., herbivore and invertivore) or high (e.g., piscivore) PPMRs leads to higher or lower cPPMRs, respectively. Here, we test whether higher cPPMRs lead to shallower size spectrum slopes for reef communities (as predicted by equation (1)). We consider reefs from warm tropical seas (coral reefs, including in the Great Barrier Reef) to cool temperate rocky reefs around the entire Australian continent, so we also consider variation associated with the large temperature gradient observed across sites. First, we establish a trait‐based model of individual‐level PPMR using 8128 individual prey size measurements from 97 common fish species, representing four broad trophic guilds (herbivores, invertivores, planktivores, and piscivores) that dominate reefs. Second, we apply the model to estimate cPPMR using empirical data on trophic guild and size structure of reef fish communities from ~15,000 underwater visual surveys around Australia. Finally, we test whether abundance size spectrum slopes, from these same survey data, are positively related with cPPMR, such that shallower slopes are generally associated with higher cPPMR, as predicted by theory (Figure 1).

## 2 METHODS

### 2.1 Sample collections and trophic guild identification

**FIGURE A1:**
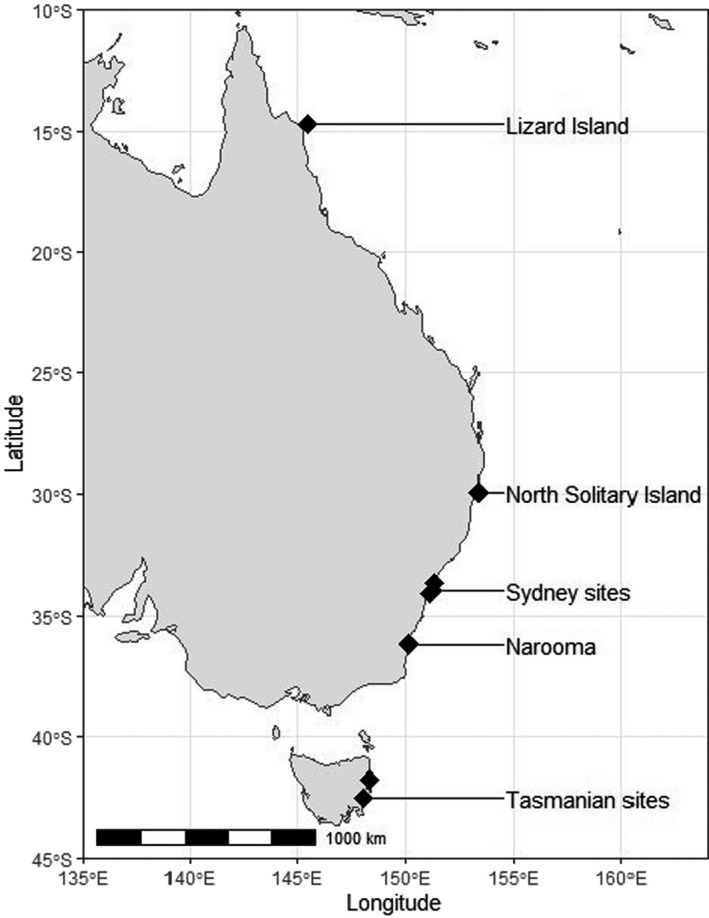
Fish collection sites, collections occurred in Spring or Autumn (2018–2019)

To assess coastal reef community cPPMR, we first characterized individual‐level predator–prey size relationships for major trophic guilds. We collected 8128 individual predator–prey measurements from the stomach contents of 325 individual fishes spanning 97 species and 1.97–7878 g in body mass, from coastal reef sites (<15 m depth) over ~30 degrees latitude along Australia's eastern seaboard (Figure A1). Fish were collected by spearfishing and placed on ice or frozen until dissection. Prior to dissection, fishes were identified to species and weighed (grams) to provide predator mass. The predominant habitat substrata at sites transitioned from rocky algal dominated reefs in the south to coral dominated reefs in the North. While the sampling scheme did not permit to sample full ontogenetic range of body sizes in each species, considerable fish size variation was sampled within each trophic guild (Table A1), which was the focus of our analyses (see below).

In general, species assemblages across ecosystems (including complex coral reefs) are characterized by a few, abundant (‘dominant’) species and a large number of comparatively rare species (Avolio et al., 2019). As the focus of this study was to explore general, trophic guild‐level PPMR values in coastal fish communities, our sampling effort concentrated on collecting individuals of locally abundant species from broad trophic guilds present at each site (Table A1). Although considerable variation in diet is known from within trophic guilds (Parravicini et al., 2020a), generalist diets are common within reef fish guilds (Van Denderen et al., 2018). Furthermore, a recent study of over 13,000 individuals from 615 fish species revealed that trophic guilds were highly conserved within families, and that body size and phylogeny alone (both included as fixed and random effects in the present study) were sufficient for predicting the trophic guild for 97% of fish in the dataset (Parravicini et al., 2020a). Therefore, research suggests that, despite the extraordinary morphological specialization of reef fish, specialized morphologies may be more indicative of ‘how’ a species eats, rather than ‘what’ they eat (Bellwood et al., 2006; Brandl et al., 2015; deVries et al., 2016). This means that the use of broad trophic guilds likely captures general feeding patterns across a range of morphologies and taxonomic levels.

Accordingly, fish species in the present study were first classified into nine trophic guilds as per Stuart‐Smith et al. (2013), then, to maximize sample sizes within each trophic guild and reduce possible misclassification error (Parravicini et al., 2020b), these classifications were further consolidated into four main trophic guilds—Herbivores: consisting of nominal and obligative herbivores; Planktivores: diets mainly consisting of zooplankton; Invertivores: omnivores, and diets mainly consisting of benthic invertebrates; and Piscivores: diets mainly consisting of large or highly mobile prey such as fish or cephalopods (Table A1). Fishes classified by Stuart‐Smith et al. (2013) as ‘Algal Farmers’ or ‘Browsing Herbivores’ and ‘Scraping Herbivores’ or ‘Excavators’ were all grouped into ‘Herbivores’; ‘Benthic Invertivores’, ‘Omnivores’, or ‘Corallivores’ were grouped into ‘Invertivores’; and ‘Higher Carnivores’ (including generalist higher predators, such as fish which feed on cephalopods) were named ‘Piscivores’. Herbivores are generally assumed to derive most of their nutrition from plant and algal material, which cannot be sized in the gut contents. However, as there were also considerable numbers of small invertebrates in the gut contents of herbivores (perhaps consumed incidentally), these were identified and measured, and the PPMR of this guild included to provide a comparison to guilds that actively target animal prey.

### 2.2 Gut content analysis and prey length–weight conversions

To assess prey sizes of the sampled fishes, we preserved and analyzed guts of fish collected. As soon as possible after field collection (or immediately upon thawing), gut contents were preserved in >70% ethanol after removal from either the stomach or the anterior alimentary canal where defined stomachs were not present (very small specimens often precluded the separation of fore and hind guts). Prey items that were sufficiently undigested to enable identification (to phylum, order, or family level for the application of length–weight conversion factors) and differentiation of the major body axis were further separated out for measurement.

**FIGURE A3:**
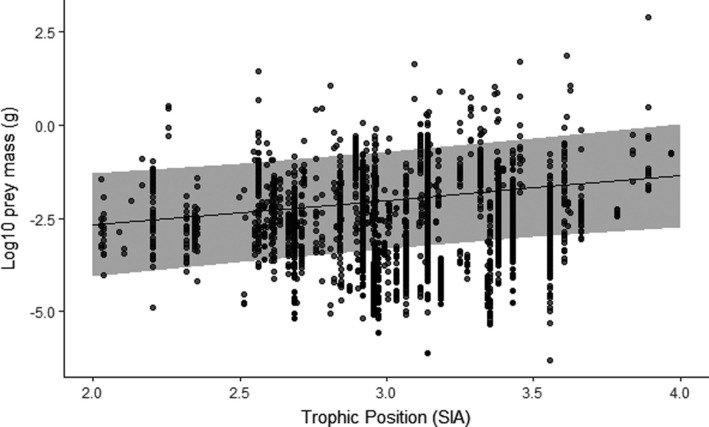
Predictions from Linear Mixed Effects model comparing individual fish Trophic Position (as calculated from nitrogen stable isotope values) and individual fish Log_10_ Prey Mass (g)

For planktivores, all prey >0.5 mm were separated from the sample, identified, and measured (majority of prey were <2 mm); for other trophic guilds, only prey >1 mm were measured due to the time‐consuming nature of the work. The difference in the minimum prey size measured applies to all planktivores evenly, however, applying the 1 mm cut‐off to this guild would likely reduce planktivore PPMR estimates. The smallest prey sizes are likely to be underestimated in all trophic guilds, as small prey are digested faster. However, we also note that our PPMR estimates are biomass weighted, meaning that small prey sizes are contributing considerably less to the cPPMR values. In cases where more than ~200 prey items per gut were present, a subsample of gut contents was measured. Where traditional standard length measurements could not be applied (e.g., barnacles, hermit crabs in shell), the longest body axis was measured (e.g., widest part of shell for barnacles). Prey items were photographed with a scale and measured using the program CPCe (Kohler & Gill, 2006) (see example Figure A3). To convert prey length measurements into body mass, prey were classified to the lowest taxonomic resolution possible and length–weight conversions from different literature sources were applied (Table A2). Where conversions are length to dry weight, a dry wet weight conversion factor described by Ricciardi and Bourget (1998) was applied.

Stable isotope data were available for a subset of the fish in this study (280 individuals) and we therefore cross‐validated the two types of data. We expected a positive relationship between prey sizes and trophic position, and such relationship was indeed found (Figure A3), suggesting that the ‘snapshot’ of species diets assessed in our study was indeed reflective of the fish's trophic position over longer time periods (see Appendix).

### 2.3 Trophic guild‐level predator–prey mass (PPMR) relationships

To quantify the relationship between predator and prey sizes and determine differences between guilds, we used weighted linear mixed effects models. All data analyses were performed using the R statistical language (R Development Core Team, 2021). We then used the package ‘lme4’ (Bates et al., 2012) to model prey mass as a function of predator mass and trophic guild identity, treating genus as a random intercept effect (after investigating alternative taxonomic random effects structures including species, family, and nested alternatives; Table A5). Furthermore, we biomass weighted the model to account for the varying contributions of small versus large prey items to a predator's energetic intake (see Reum et al., 2019).

Predator mass was considered an independent variable as directly measured, whereas each unique fish number (predator ID) was treated as a random effect to account for the repeated measures of prey items for one predator. To account for any phylogenetic influences on prey size arising from unmeasured aspects of foraging behavior, we included a nested random effects term ‘Genus’ in our model, within which an individual fish (ID) was nested (‘Genus/ID’). Different fixed and random effects structures were compared (Table A4 and A5), and although all differences in the Akaike information criterion (AIC) values were below 4 (Table A4), the model with the lowest (AIC) value contained the fixed effects of predator mass (log_10_ transformed; continuous), trophic guild (categorical), and their interaction, and the nested, random effects termed ‘Genus/ID’:

(2) $$Y_{i}=\beta_{0}+{{\mathrm{wt}}_{i}\beta }_{1}M_{j}+\beta_{2,T}T_{j}+\;\beta_{3,T}M_{j}*T_{j}+\alpha_{\mathrm{Genus}:\mathrm{ID}\left[j\right]},$$

where $Y_{i}$ is the log_10_ transformed individual prey mass, $M_{j}$ is the log_10_ transformed individual predator mass, $T_{j}$ is the categorical value defining predator's trophic guild, $\alpha_{\mathrm{Genus}:\mathrm{ID}[j]}$ is the random intercept effect, and *β*_0_, *β*_1_, *β*_2,T_, and *β*_3,T_ are body mass, trophic guild, and interaction coefficients to be estimated. Restricted maximum likelihood (REML) was applied to all models, and residual and Q‐Q plots were checked to ensure sufficient concordance with model assumptions. In order to weight the individual prey items via total prey biomass, a weighting term, ${\mathrm{wt}}_{i},$ was added to the model (in lme4, syntax: weights = wt). We calculated PPMR by dividing the model prey size predictions (with and without the random effects) by the predator mass, and visualized the outcomes (as per Barnes et al., 2010; see Figure 3 below).

### 2.4 Community‐level PPMR estimates

Community‐level PPMR (cPPMR) was obtained by averaging the individual PPMR of all predators within a given study area (Nakazawa, 2017; Reum et al., 2019b). Calculating a cPPMR requires information on the sizes of the individual predators and their prey in size class bins (Blanchard et al., 2017; Nakazawa, 2017; Reum et al., 2019b). Data on the size ranges and abundances of the four trophic groups in coastal communities came from underwater visual censuses on shallow reefs by the Reef Life Survey (RLS) and Australian Temperate Reef Collaboration (ATRC) programs (Edgar & Barrett, 2012; Edgar et al., 2020; Edgar & Stuart‐Smith, 2014). The RLS and ATRC data, accessed through the Integrated Marine Observing System's National Reef Monitoring Network facility (https://portal.aodn.org.au/ search, Accessed 21/08/2020), include the abundance and size classes of all fish species observed within 500 m^2^ belt transects on shallow rocky and coral reefs (for details on underwater transect methods, see Edgar & Barrett, 2012; Edgar et al., 2020; Edgar & Stuart‐Smith, 2014). Only transects from Australia surveyed from 2007 onwards, with biomass estimates available for all species, were included; resulting in a total of 14,941 transects.

All fish in the visual survey dataset were classified into the four trophic guilds, as described above. Next, using the linear mixed effects model, prey mass was estimated for each individual observed fish in the survey, using the fish's wet mass (g), estimated from the observed length, and its trophic guild identity. Nearly, all fish surveyed could be categorized into one of the four broad trophic guilds, however, species classified as ‘cleaners’ (e.g., cleaner wrasse), along with some non‐fish predators (marine mammals, reptiles, and birds), were excluded from the dataset. With these data, we then calculated transect‐level PPMR (cPPMR) by summing the PPMRs of fish in each trophic guild and size class combination, and dividing by the total number of individuals observed:

(3) $$cPPMR=\frac{\sum_{i=1}^{n}\sum \left({\mathrm{PPMR}}_{i,M}*N_{i,M}\right)}{\sum_{i=1}^{n}N_{i,M}}$$

where *PPMR_i_*_,_ *_M_* is the estimated PPMR value of trophic guild *i* at size group *M*, and N*_i_*_,_ *_M_* is the number of individuals observed. To determine the sensitivity of the cPPMR metric to the exclusion of trophic guilds, we tested the resulting values by excluding one trophic guild at a time, and re‐running the calculation (Table A6, Figure A5). Only the exclusion of invertivores had a large impact on the resulting cPPMR; however, as we are interested in the whole community, all trophic guilds were included in subsequent analyses.

### 2.5 Size spectrum models

The term ‘abundance size spectrum’ refers to the relationship between body size (e.g., mass) and abundance, and is often represented on the log–log scale, that is, the logarithmic abundance of individuals within logarithmic body size classes (Figure 1). Here, we used Australian rocky and coral reef community abundance size spectrum slopes (equivalent to *b*−1 in terms of biomass size spectrum slopes) from Heather, Blanchard, et al. (2021), where slopes were estimated by fitting a linear mixed effects model with log abundance as the response variable, log body size class as a fixed predictor variable, and with site nested within ecoregion as random predictor variables. The community size spectra data derived from RLS transects used in the present study included both fish and invertebrate size and abundance data. As assessed in Heather, Blanchard, et al. (2021), excluding invertebrates from community size spectrum data can lead to a spurious interpretations. As discussed in Heather et al. (2021), the inclusion of the smallest body size classes of fish and invertebrates (<32 g) in diver surveys have been questioned due to possible methodological issues influencing survey observations. However, the authors recommended the inclusion of both invertebrates and the smallest body size classes as the reduced abundance of the smallest individuals observed in these size spectra may be a true component of the underlying body size distribution (as discussed in Heather, Stuart‐Smith, et al., 2021). We use the complete fish and invertebrate dataset for our analysis as the predators sampled consumed both small‐sized and invertebrate prey, and truncating the dataset at fish >32 g (removing all fish smaller than 13–16 cm) would exclude a vast majority of planktivores, resulting in a biased representation of the community.

### 2.6 Relationship between cPPMR and size spectrum slope

Size‐based theory predicts that animal communities with higher cPPMRs will have shallower size spectrum slopes, which means they will have a relatively greater number of large‐bodied individuals than communities with low cPPMRs. To test this hypothesis, we linearly regressed our estimated cPPMR from each transect to fish and invertebrate community slope values estimated for the same transects by Heather, Stuart‐Smith, et al. (2021). We applied a linear mixed effects models in the R language package ‘lme4’ (Bates et al., 2012). The RLS‐ATRC sites in our dataset were surveyed over a range of years and span a large spatial temperature gradient from annual mean sea surface temperatures (SST) of ~14°C up to ~28°C, so we included annual mean SST as either a fixed or random intercept effect (Table 1, Table A7). Mean SST data were derived from Bio‐ORACLE (Tyberghein et al., 2012) and matched to RLS‐ATRC sites. We further included site nested in year as random intercept effects, given that some sites were repeat sampled over multiple years. We also ran the model excluding the 1st and 99th percentiles of cPPMR to determine if our results were sensitive to extreme values of cPPMR caused by unusual fish composition at a site (Table A9).

**TABLE 1:** Linear mixed effects statistics for the model used to predict fish community size spectrum slope (*b*−1), according to log_10_ cPPMR and mean Sea Surface Temperature (Mean SST; °C). Fixed effects: log_10_ cPPMR (continuous) and Mean SST (continuous, °C). Random effects: site (as multiple transects were sometimes conducted at the same site within the same year) and year (some sites were repeatedly sampled over years). Model syntax in package: lmer (*b* ~ cPPMR * Mean SST + (1|Year/Site), REML = T). ‘Drop1’ analysis of the model's structure revealed the interaction term of the model could not be dropped without significant effects on the model output (*p* < .001)

Predictors	Size spectrum slope		
	Estimates	CI	*p*
(Intercept)	1.67	1.03 – 2.31	**<.001**
Log_10_ cPPMR	−0.40	−0.56 – −0.23	**<.001**
Mean SST	−0.13	−0.16 – −0.10	**<.001**
Log_10_ cPPMR * Mean SST	0.03	0.02 – 0.04	**<.001**
*Random effects*			
σ^2^	0.03		
τ_00 Site:Year_	0.02		
τ_00_ Y_ear_	0.00		
ICC	0.39		
*N*_site_	1220		
*N*_mean SST_	11		
Observations (transects)	5401		
Marginal *R* ^2^ / Conditional *R* ^2^	0.197 / 0.508		

## 3 RESULTS

### 3.1 Establishing predator–prey mass ratios from individual‐scale measurements

**FIGURE 2:**
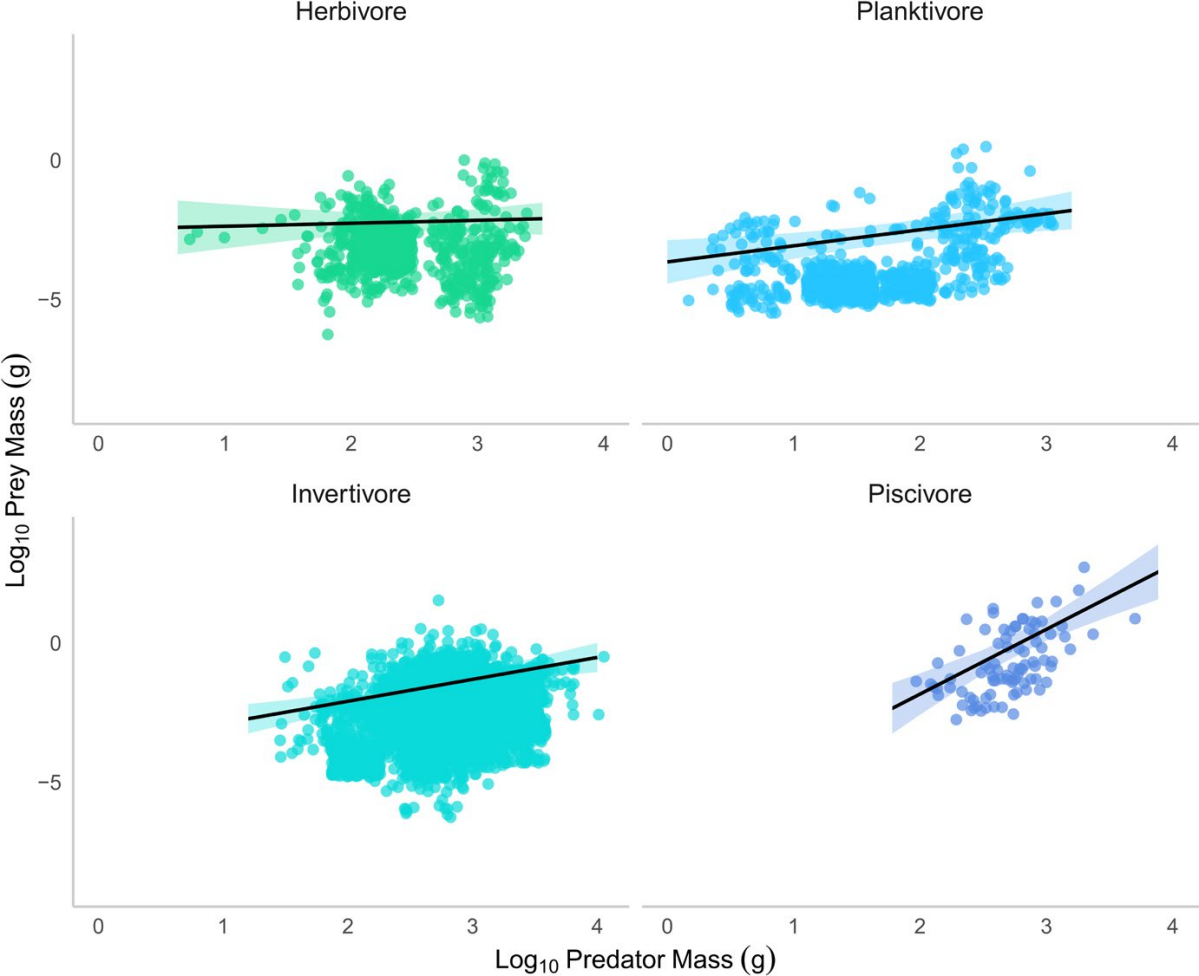
Predator–prey mass relationships. Predictions and 95% confidence intervals generated by linear mixed effects model for prey mass as a function of predator mass (log_10_, g) and trophic guild. The regression lines of the model reflect the biomass weighting of individual prey within a predator, while data points represent prey items from the gut contents of individual fishes (see Table A1 for species assigned to each trophic guild). Marginal and conditional *R^2^* for the model were 0.33 and 0.995 (note: individual was included as a random effect)

**FIGURE A4:**
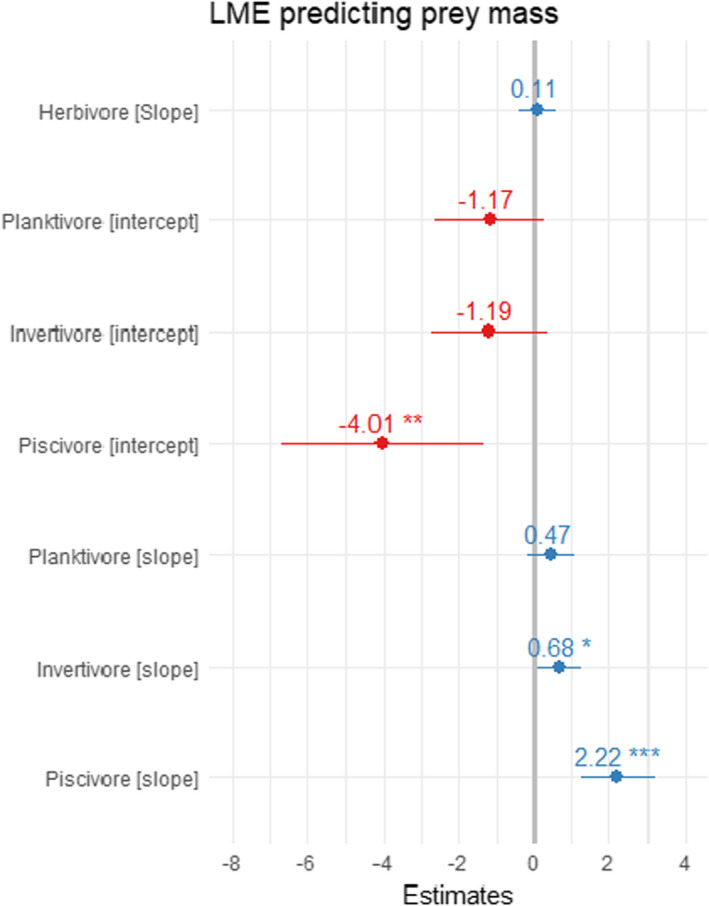
Estimates of the Linear Mixed Effects (LME) model used to predict log_10_ prey mass (g), term's significance indicated by asterix (*<.05; **<.01; ***<.001)

**FIGURE A5:**
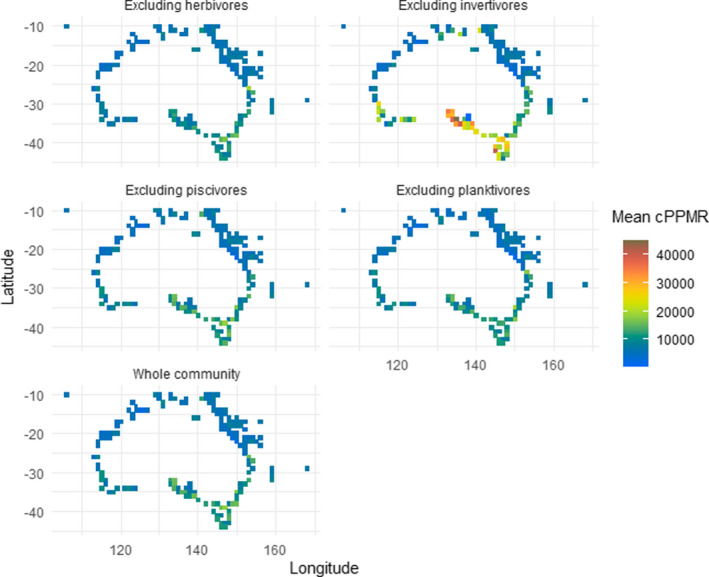
Testing the effect of excluding individual trophic guilds (one at a time) on the resulting cPPMR. Only the exclusion of the invertivore fish guild appears to have a clearly notable impact on the cPPMR of southern sites, whilst minor changes in the cPPMR are observable in each of the other scenarios, however the overall trend remains the same. With invertivores excluded the trend observed for the ‘whole community’ is amplified rather than contradicted (i.e., the mean cPPMR in the south increases)

Of the 992 fish individuals collected by the study (148 species), 325 fish had non‐empty stomachs with sizeable prey items (97 species). For these fish, prey sizes ranged from 0.12 to 189.59 mm (Table A1). The mixed‐effect model analysis showed that for all fishes sampled, ~33% of the variation in the measured prey mass (marginal *R* ^2^) could be explained by the two predictor variables (body size and trophic guild) and their interaction (Figure A4, Table A3). The models showed that prey mass increased with the predator mass (significantly positive slopes, Figure 2, Table A3) in three of the four trophic guilds. These slopes were steepest in piscivores (Figure 2, Table A3; slope: 2.22; *p* < .001), shallower but still significantly positive in invertivores (slope: 0.68, *p* = .025), positive although not significant in planktivores (slope: 0.47, *p* = .14), and not significant in herbivores (slope: 0.11; *p* = .664; Table A3).

**FIGURE 3:**
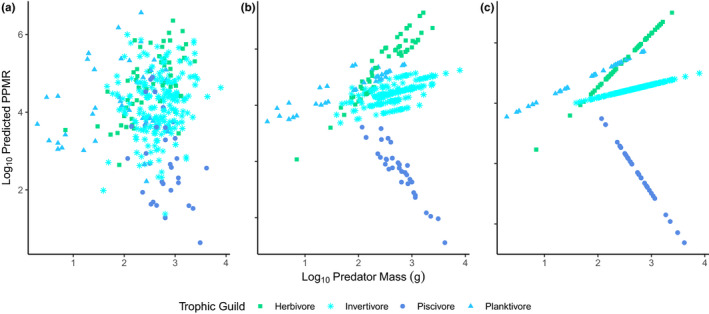
Predictions of the linear mixed effects model with the fixed effects: Trophic Guild and Log_10_ Predator Mass (g); and the random effects individual fish nested in Genus. Predictions are shown based on: (a) including random effects of genus and individual; (b) including only random effect of genus and excluding the effects of individual; and (c) fixed effects only (trophic guild)

To explore the implications of the nested random effects structure and visualize the general relationship between predator size and its PPMR in each of the four trophic guilds (as per Barnes et al., 2010), we show a range of model predictions using a full and simplified random‐effect structure (Figure 3). By deriving the predicted PPMR using the model predictions of prey mass (log_10_ (g)) and the individual weight of predators (log_10_ (g)), we show the extent to which random and fixed effects contribute to the model's predictions. Here, predictions are converted into PPMR (rather than prey mass) versus predator mass, to make them more comparable to Barnes et al.’s (2010) analyses. These visualizations show, as was also the case in Barnes et al. (2010), that a large proportion of variation is accounted by individual‐level variation across predators (Figure 3a). Nevertheless, both removing random individual effects but maintaining the taxonomic groupings (genus‐level random effects; Figure 3b) and reducing the model predictions to the four trophic guilds (no random effects; Figure 3c) provide a similar trend between PPMR and predator's body size. Importantly, this relationship reveals that PPMR values are not fixed across predators’ body sizes. For instance, steep increases in PPMR with body size are apparent for herbivores (i.e., larger fish feed on relatively smaller prey), in contrast, steep decreases in PPMR with body size are apparent for piscivores (i.e., large predators feed on relatively larger prey). As we were interested in broad‐scale estimates at the trophic guild level, we did not propagate error from individuals or other random effects up to the cPPMR level, but note that the random effects introduced through genus, as included in the model, still produced the same visual pattern as the fixed effects alone.

### 3.2 Relationship between community‐level PPMR and size spectrum slopes

Scaling the measured relationships between fish and prey sizes up to the community data from reef fish surveys revealed an overall mean community PPMR (cPPMR) of ~8700 (across‐site variation: minimum 21; first quartile 5751, median 8305; and third quartile 12,507, maximum 15,776,588). This overall mean takes into account the trophic guild composition and size structure observed in Australian coral and rocky reef fish communities.

**FIGURE 4:**
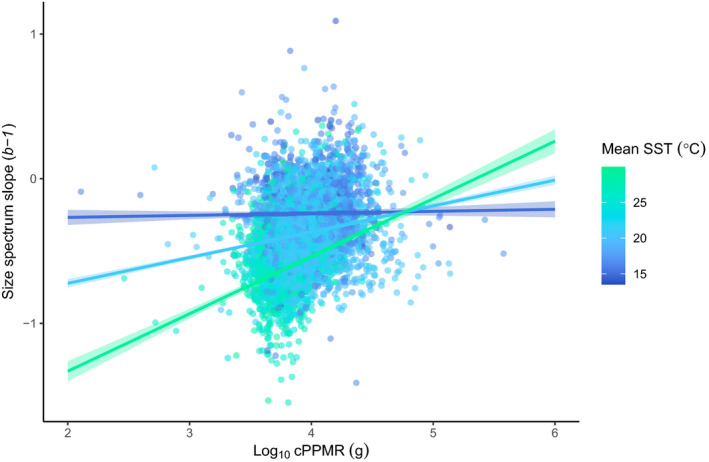
Relationship between fish and invertebrate abundance size spectrum slope (*b*−1) and log10 cPPMR values for fish communities

As predicted by equation (1), communities with higher cPPMRs possessed shallower size spectrum slopes (thus including a greater proportion of large‐bodied individuals, than communities with low cPPMRs), when using the full fish and invertebrate size spectrum slope dataset. We found a significant, positive, relationship between log_10_ cPPMR and abundance size spectra slope both when including (*p* < .001; Marginal *R* ^2^/Conditional *R* ^2^ 0.20/0.51; Table A1) and excluding (*p* < .001; Marginal *R* ^2^/Conditional *R* ^2^ 0.03/0.54; Table A7) mean sea surface temperature (mean SST) as an explanatory variable, interacting with cPPMR. When cPPMR was removed from models aiming to predict size spectrum slope from SST alone, the model predictive power was considerably weaker (delta AIC > 150). The model with cPPMR interacting with mean SST had the greatest explanatory power of all possible predictor combinations (Table A8, delta AIC ~35) and showed that the relationship between cPPMR and community size spectrum slope *b*−1 was strongest in the tropics, weakening towards cooler temperatures (interaction term *p* < .001, Table 1, Figure 4) and close to no relationship in the coolest, temperate Tasmanian sites (Figure 2).

**FIGURE A6:**
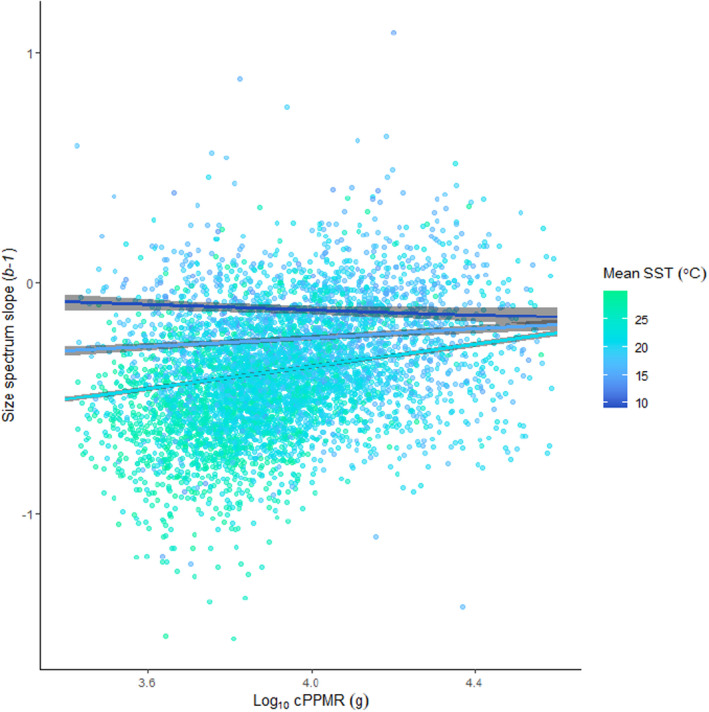
Relationship between size spectrum slope (*b*−1) and log_10_ cPPMR values for fish communities, excluding 1st and 99th percentiles of cPPMR

Predictions and confidence intervals of linear mixed effects model for abundance size spectrum slope as a function of log_10_ cPPMR (g) and temperature (mean annual SST, °C), with site and year as random effects (see Figure A6 for the data excluding the 0.01 and 0.99 quantiles; direction and significance of predictions are the same). Marginal and conditional R*^2^* for the model were 0.20 and 0.51. Data points represent fish communities per individual RLS transect. To visualize the interaction effect, trendlines are provided for the three temperature values corresponding to averages in Tasmania, New South Wales, and Queensland (15, 21, and 29°C). Confidence intervals (ribbons either side of lines) for the interaction prediction lines likely underestimate the compound error as they represent one discrete value of a continuous variable (temperature).

Results were similar in direction, significance, and resulting marginal and conditional *R* ^2^ values when the same analysis was run with the 1st and 99th percentile cPPMRs excluded (removing 109 transects from the dataset; Table A9), suggesting the results are rigorous to the removal of cPPMR outliers (i.e., transects dominated by small or large fish aggregations, Figure A6).

## 4 DISCUSSION

Our study presents evidence for the key role of cPPMRs in size structuring reef fish communities. In doing so, we provide four main findings: (1) PPMR of reef fishes varies differently with body size both between and within trophic guilds; (2) cPPMR of Australian rocky and coral reef fishes appears considerably higher than has been assumed by most modeling studies; (3) a significant positive relationship exists between reef abundance size spectra slopes and cPPMR, suggesting that cPPMR partly explains variation in natural size structure of reef communities; and (4) the relationship between cPPMR and size spectrum slopes strongly depends on temperature.

### 4.1 High community PPMR values in coastal Australian reefs

We found that PPMR increased with increasing body size across three trophic groups (piscivores, invertivores, and planktivores), but decreased with body size in herbivores (the only trophic guild assumed to not actively target animal prey; Figure 3). Increasing PPMR with body size is consistent with previous studies (Edgar & Shaw, 1995; Griffiths, 2020; Niiranen et al., 2019; Reum & Hunsicker, 2012; Scharf et al., 2000; Wilson & Kimmel, 2022), including the general positive trend across an extensive dataset of fish and squid (regardless of trophic guild) found using gut content analysis by Barnes et al. (2010). While specific PPMR values may be applied to different predators (both at the species, Andersen, 2019; and trophic guild levels, Reum et al., 2019a), treating these PPMR values as constant regardless of body size is common practice in size‐based ecosystem models (likely due to the paucity of data). A positive relationship between PPMR with body size may have considerable implications for energy transfer in ecosystems, as it suggests large‐bodied predators continue to receive energy from small‐bodied (Griffiths, 2020; Scharf et al., 2000; Tsai et al., 2016), lower trophic‐level prey, thus losing less energy through transfer inefficiencies (Barnes et al., 2010) and facilitating higher abundances of these predators at large body sizes. Our results suggest that holding PPMR estimates constant regardless of predator body size may mislead predictions from size‐based ecosystem models, including when scaling up to the community level.

Community‐level PPMR integrates taxonomic composition, size distributions, and feeding preference into one ecologically important summary statistic (Brose et al., 2019). Yet, despite being a valuable metric, estimation of cPPMR values remains rare and varies greatly across species and ecosystems (Brose et al., 2006, 2019). This study provides the first approximate estimate of community predator–prey mass ratios (cPPMRs) for shallow eastern Australian rocky and coral reefs and shows that mean values observed (~8700) are similar to those found by stable isotope analysis (SIA) for a tropical reef system (Al‐Habsi et al., 2008), but up to ~20 times higher than previous SIA‐based studies for open shelf and other temperate and tropical reef systems (Jennings & Blanchard, 2004; Trebilco et al., 2016; Zhu et al., 2019).

Several methodological reasons may explain the variable cPPMR values observed across studies (beyond ecosystem type; Nakazawa, 2017; Reum et al., 2019b; Tsai et al., 2016). First, some of the differences between our results and other studies may be because all other marine cPPMR estimates were based on SIA, whereas we used direct prey size analyses from stomach contents. The advantage of SIA is that it integrates information on species diets over longer timeframes (days to years depending on the tissue type; Boecklen et al., 2011; Jennings et al., 2008; Nielsen et al., 2018; Vanderklift & Ponsard, 2003) and is less prone to random spatial and temporal variation. However, a major challenge is that currently most SIA‐derived cPPMR estimates are based on ‘raw’, non‐baseline–corrected δ^15^N values, and therefore do not account for differences in isotopic baselines between locations. We also collected δ^15^N data and observed large differences in isotopic baselines across our sampled locations, which suggests that deriving PPMR estimates using the common SIA approach would be misleading. Nevertheless, the δ^15^N data available for a subset of the fish analyzed here (280 individuals) generally confirmed the robustness of our dietary analyses. The expected positive relationship between prey sizes and trophic position (Jennings et al., 2002) was indeed evident in our samples (Figure A3), suggesting that the ‘snapshot’ of species diets assessed in our study using gut contents was reflective of the fish's trophic position over longer time periods. Additionally, we note that our results are in the same magnitude as a study using SIA on reef fish communities (Al‐Habsi et al., 2008).

Another possible reason for differences in our cPPMR estimates to those from other reef studies (namely Trebilco et al., 2016; Zhu et al., 2019) is that our limited sample sizes prohibited accurate evaluation of ontogenetic and spatial trends in PPMR within species, although these likely occur. Gut content analysis typically exhibits a high degree of noise due to samples providing a brief snapshot of a fish's diet (Nielsen et al., 2018). Yet, the focus of this study was not on the type of prey, but the size of prey, within broad trophic guilds, and the statistical models used here aimed to account for random variation associated with individual, its genus, and sampling sites. Earlier studies have shown that predator size alone is an important predictor of prey sizes (Soler et al., 2016) and here we refine this prediction with addition of four trophic guilds. Thus, despite the possible methodological issues, we believe that our results show genuinely high cPPMR values for coastal reef communities.

The high cPPMR values observed for reef versus pelagic or shelf habitats (such as Jennings & Blanchard, 2004) could be explained by the high degree of structural complexity in these habitats, which provide abundant refuges for a range of predator and prey sizes (Brose et al., 2019; Wang et al., 2009). While refuges ‘lock’ some prey away from predation, both prey and their predators are more abundant when refuges are present (Hixon & Beets, 1993); and habitat complexity only provides refuges for prey up until either the point of refuge saturation, or when prey themselves must exit the refuge to forage (Donelan et al., 2016). More complex habitats could therefore provide a stable trickle of prey (Rogers et al., 2014, 2018), allowing reef fishes to feed on relatively small, and sub‐optimal, prey sizes (Griffiths, 2020; Portalier et al., 2019). Empirical studies have shown that even while maximum and mean prey size usually increase with predator size, small prey often continue to be consumed (Floeter & Temming, 2003, 2005; Gaeta et al., 2018; Juanes & Conover, 1994; Ménard et al., 2006). This hypothesis could be addressed in future studies by adding habitat complexity metrics as additional predictors of variation in cPPMR.

Furthermore, in coastal ecosystems, multiple, largely independent, sources of primary production may provide alternative food supply sources (i.e., benthic and pelagic, unicellular and macrophytic) (Trebilco et al., 2016). Along with the nutritional and structural components of benthic primary productivity on reefs, planktonic primary productivity is well recognized as a substantive contributor to reef fish trophodynamics (Bray et al., 1981; Hamner et al., 1988; Holland et al., 2020; Morais & Bellwood, 2019; Odum & Odum, 1955; Polunin, 1996; Truong et al., 2017; Wyatt et al., 2012). The turnover of benthic and planktonic primary producers generally operates over different timescales, and can therefore fuel reefs through contrasting perturbations, stabilizing the supply of energy higher up the food chain (Rooney et al., 2006). In a global study of marine teleosts, generalist diets were found to be favored over specialist where benthic and pelagic sources both contribute to primary productivity, opposed to pelagic only pathways (Van Denderen et al., 2018). In summary, by providing alternative, persistent, and alternative sources of primary production, rocky and coral reef ecosystems may enable consumption of smaller, less energetically rich prey, facilitating the establishment of high PPMRs.

### 4.2 Community PPMR and temperature are important predictors of size spectrum slopes

Our study shows that both cPPMR and site temperature (here measured as mean annual sea surface temperature) explain some variation in community size spectrum slopes (*b*−1) and that there is significant interaction between these factors. A recent study (Heather, Blanchard, et al., 2021) showed that abundance‐based size spectrum slopes around Australia varied considerably around the theoretical expected mean of −1 (although our slopes are shallower, as unlike Heather, Blanchard, et al., 2021, we included the full range of sizes observed). A large body of literature has demonstrated that temperature is an important predictor of community size spectrum slope and that these slopes are usually steeper in higher temperatures (Heneghan et al., 2019; Pomeranz et al., 2022). Here, we show that cPPMR also explains a significant amount of variation in size spectrum slopes, but not in cool temperate areas. In tropical areas, the relationship between size spectrum slope and cPPMR was quite steep, whereas in cool temperate reefs, it was close to 0 (Figure 4). There are several possible reasons for this interaction.

The relationship between size spectrum slopes (*b*−1) and the cPPMR–mean SST interaction may be a consequence of our cPPMR data not including invertebrate PPMRs. As described in Heather, Blanchard, et al. (2021), excluding either the invertebrate or fish components of a community may result in misleading patterns, as the relative contribution of invertebrates to overall community composition and trophic ecology is greater in marine systems at higher latitudes with lower mean SSTs. Furthermore, our study may not have fully captured temperature‐related differences in trophic guild PPMR, as the limited sample size precluded detailed comparisons across temperatures. If individual fish PPMRs change with temperature, our cPPMRs would also change, possibly explaining more of the size spectra slopes in temperate ecosystems. Yet, it is also possible that at colder temperatures, the other term in equation (1)—trophic transfer efficiency (TE)—is more variable and provides a compensatory role. A tight relationship between cPPMR and size spectrum slope would assume that TE is similar across sites, while highly variable TE would randomize the relationship between *b*−1 and cPPMR. A recent review revealed considerable variation in estimates of the mean value of trophic transfer efficiency (TE) globally, with a general trend of higher estimated TE in colder ecosystems (Eddy et al., 2021).

Finally, systematic variation in size spectrum slopes could be explained by human impacts (e.g., fishing and pollution) or environmental differences that were not accounted for in the slopes used here (Dulvy et al., 2004; Graham et al., 2005; Nash & Graham, 2016; Robinson et al., 2017); however, we note that many of our sites and species are lightly exploited by global standards (see Audzijonyte et al., 2020). Understanding and disentangling the nature of these influences requires improved site‐level covariate data to investigate factors contributing to interactions in greater detail.

In conclusion, our study—the first general estimate of cPPMRs across Australian coastal reef communities—revealed mean cPPMR values up to threefold higher than many previous estimates, but consistent with values observed for a tropical reef (Al‐Habsi et al., 2008). This finding has considerable implications for size‐based models, which are currently based on limited and variable estimates for cPPMR in marine systems. By providing empirical estimates of cPPMR for this system, this study may improve our capacity to predict changes in reef fish community structure, and its responses to human and environmental pressures.

## CONFLICT OF INTEREST

The authors declare no competing interests.

## AUTHOR CONTRIBUTIONS

**Amy Rose Coghlan:** Conceptualization (supporting); Data curation (lead); Formal analysis (lead); Investigation (lead); Methodology (equal); Project administration (lead); Resources (supporting); Validation (equal); Visualization (lead); Writing – original draft (lead); Writing – review & editing (lead). **Julia L. Blanchard:** Conceptualization (equal); Data curation (equal); Formal analysis (equal); Funding acquisition (equal); Investigation (equal); Methodology (equal); Project administration (equal); Supervision (equal); Validation (equal); Writing – original draft (equal); Writing – review & editing (equal). **Freddie J. Heather:** Data curation (equal); Formal analysis (equal); Validation (equal); Writing – review & editing (supporting). **Rick D. Stuart‐Smith:** Conceptualization (equal); Data curation (equal); Formal analysis (supporting); Funding acquisition (equal); Investigation (supporting); Methodology (equal); Project administration (supporting); Supervision (equal); Validation (supporting); Writing – original draft (equal); Writing – review & editing (supporting). **Graham J. Edgar:** Data curation (equal); Formal analysis (supporting); Investigation (supporting); Methodology (equal); Project administration (equal); Writing – original draft (supporting); Writing – review & editing (supporting). **Asta Audzijonyte:** Conceptualization (equal); Data curation (equal); Formal analysis (equal); Investigation (equal); Methodology (equal); Project administration (equal); Supervision (equal); Validation (equal); Writing – original draft (equal); Writing – review & editing (equal).

## ETHICAL APPROVAL

Ethics approval for this project was granted by the University of Tasmania Animal Ethics Committee (A0017225).

## Supporting information

SupInfo

## Figures and Tables

**FIGURE A2:**
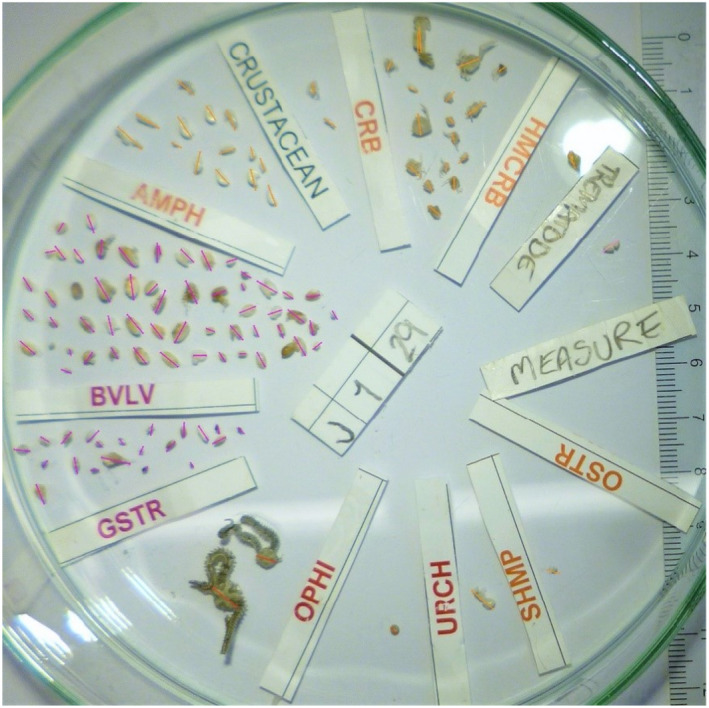
Measurable (see methods) gut content items laid out for analysis. Items are grouped into broad categories and photographed with a scale, before being processed for measurements using the program CPCe (coloured lines indicate measurement axis). Label in centre indicates the individual fish from which items were obtained, in this case a *Cheilodactylus spectabilis* individual (fork length 350 mm, wet weight 769 g, collected from Narooma, NSW)

## Data Availability

Underwater visual survey abundance datasets are available through the Integrated Marine Observing System's National Reef Monitoring Network facility (https://portal.aodn.org.au/search). The slope calculation datasets are available from Heather, Blanchard, et al. (2021), Heather, Stuart‐Smith, et al. (2021). The gut content analysis datasets used in this analysis and all R code is available through the code repository at https://github.com/amroco/Reef_fish_cPPMR.

## 1 Specimen collection & gut content analysis

Fish specimens used in this study were opportunistically collected from a variety of sites along a latitudinal gradient (Figure A1), as part of a larger study investigating stable isotope values in reef fish tissues. Although a total of 876 individual fish (from 140 species, 83 genera, and 37 families) were sampled, only 325 individuals (from 97 species, 61 genera, and 34 families) contained ‘measurable’ (see methods for definition) gut content items, with species further categorised into trophic guilds (Table A1). Meanwhile, prey items from these fish were measured and lengths converted to weights as per the methods (also see Figure A2) and Table A2.

**TABLE A1:** Specimens collected for dietary analysis, subsequently classified within to four trophic guilds (Herbivore, Planktivore, Invertivore, Piscivore) for analysis. Note: only prey that were both identifiable and sufficiently intact to enable measurement were included in the study, and in some cases prey items were subsampled and multiplied accordingly. The number of individuals and prey items per trophic guild are summarised

Species	*N* predator	Mean predator mass (g)	*N* prey measured per species	Mean prey mass (g)
**Herbivore**				
*Acanthurus dussumieri*	4	795.64	119	6E−04
*Acanthurus nigrofuscus*	1	179.00	1	0.001
*Acanthurus olivaceus*	3	305.00	11	0.002
*Acanthurus xanthopterus*	4	1337.71	21	0.002
*Aplodactylus arctidens*	1	1300.00	4	0.015
*Aplodactylus lophodon*	1	824.00	1	0.009
*Chironemus marmoratus*	1	134.50	1	2E−04
*Ctenochaetus striatus*	1	206.00	2	4E−04
*Dischistodus perspicillatus*	3	138.57	7	0.006
*Girella elevata*	1	1287.00	1	0.006
*Girella tricuspidata*	3	1117.67	36	0.173
*Girella zebra*	1	760.00	3	5E−04
*Kyphosus* spp.	1	2432.00	1	0.012
*Kyphosus sydneyanus*	1	1000.00	2	0.076
*Parma microlepis*	11	157.92	479	0.004
*Parma unifasciata*	2	147.91	4	0.013
*Prionurus maculatus*	3	1306.00	28	0.003
*Prionurus microlepidotus*	4	196.07	14	0.016
*Siganus corallinus*	1	219.00	2	0.008
*Siganus fuscescens*	3	221.33	3	0.006
*Siganus lineatus*	5	585.92	13	0.006
*Stegastes apicalis*	2	60.50	2	0.014
	Summary			
	*N* prey items	754	Min. mass (g)	7
	*N* individuals	56	Max. mass (g)	2432
**Planktivore**				
*Acanthochromis polyacanthus*	1	19.00	9	6.57E−05
*Atypichthys strigatus*	11	24.79	974	3E−04
*Caesio cuning*	4	267.17	24	0.11
*Heniochus* spp.	1	235.50	3	0.002
*Myripristis adusta*	2	187.75	12	0.003
*Pempheris* spp.	3	67.75	20	0.05
*Schuettea scalaripinnis*	2	77.49	143	4.95E−05
*Scorpis aequipinnis*	4	462.11	65	0.017
*Scorpis lineolata*	9	276.05	57	0.098
	Summary			
	*N* prey items	1312	Min. mass (g)	1.97
	*N* individuals	39	Max. mass (g)	69.3
**Invertivore**				
*Acanthaluteres vittiger*	5	232.92	38	0.006
*Acanthopagrus australis*	5	395.72	18	0.153
*Chaetodon flavirostris*	2	130.95	21	0.005
*Cheilinus fasciatus*	3	399.25	8	0.025
*Cheilodactylus fuscus*	29	789.98	1676	0.016
*Cheilodactylus spectabilis*	10	1245.98	1864	0.008
*Choerodon schoenleinii*	1	2338.00	1	0.003
*Cnidoglanis macrocephalus*	1	1656.00	8	0.356
*Coris gaimard*	1	73.00	3	0.002
*Coris picta*	1	210.00	1	0.06
*Dascyllus aruanus*	2	4.60	5	2E−04
*Diagramma labiosum*	2	1027.11	54	0.021
*Enoplosus armatus*	5	129.54	324	0.005
*Eubalichthys mosaicus*	8	827.96	36	0.012
*Gymnocranius* spp.	4	899.82	34	0.037
*Halichoeres chloropterus*	3	62.18	11	0.012
*Hemigymnus melapterus*	5	469.07	42	0.004
*Lethrinus harak*	5	314.42	19	0.058
*Lethrinus nebulosus*	3	1567.86	21	0.049
*Lethrinus* spp.	1	559.00	2	0.079
*Meuschenia australis*	1	278.00	4	0.006
*Meuschenia freycineti*	6	660.55	198	0.156
*Meuschenia trachylepis*	7	348.81	367	0.004
*Microcanthus strigatus*	1	26.00	11	2E−04
*Nemadactylus douglasii*	1	547.00	268	0.004
*Notolabrus fucicola*	3	595.56	16	0.005
*Notolabrus gymnogenis*	14	845.89	389	0.013
*Notolabrus tetricus*	3	276.00	13	0.01
*Ophthalmolepis lineolatus*	9	218.73	22	0.046
*Parupeneus barberinus*	9	477.54	46	0.763
*Parupeneus ciliatus*	1	103.00	1	0.008
*Parupeneus indicus*	3	300.76	25	0.137
*Parupeneus spilurus*	8	648.63	82	0.077
*Plectorhinchus albovittatus*	1	7878.00	6	0.072
*Plectorhinchus chaetodonoides*	6	2114.95	110	0.018
*Plectorhinchus chrysotaenia*	1	1192.00	32	0.002
*Plectorhinchus flavomaculatus*	3	2266.76	37	0.048
*Plectorhinchus gibbosus*	2	4059.65	23	0.099
*Plectorhinchus lineatus*	2	1768.67	15	0.183
*Pomacanthus sexstriatus*	1	675.00	2	0.002
*Pomacentrus moluccensis*	1	1.97	3	2E−04
*Pomacentrus* spp.	1	3.34	6	0.001
*Sargocentron spiniferum*	1	464.00	1	2.227
*Scolopsis bilineata*	1	74.00	1	0.003
*Scolopsis margaritifer*	1	290.00	13	0.02
*Scolopsis monogramma*	6	395.14	50	0.01
*Scorpaena jacksoniensis*	3	484.33	3	0.34
*Sufflamen chrysopterum*	4	120.72	46	0.004
*Thalassoma lutescens*	1	358.00	1	0.002
	Summary			
	*N* prey items	5974	Min. mass (g)	39
	*N* individuals	197	Max. mass (g)	886
**Piscivore**				
*Acanthistius ocellatus*	8	855.50	22	34.59
*Aulopus purpurissatus*	1	1161.00	1	7.635
*Aulostomus chinensis*	1	430.00	8	0.093
*Carangoides fulvoguttatus*	2	592.40	10	1.139
*Carangoides plagiotaenia*	1	644.00	4	0.24
*Caranx papuensis*	2	1666.00	2	34.91
*Cephalopholis cyanostigma*	3	366.33	3	6.389
*Dinolestes lewini*	4	411.36	14	1.206
*Epibulus insidiator*	1	545.00	1	1.95
*Epinephelus malabaricus*	1	4139.00	1	11.19
*Epinephelus merra*	1	117.00	1	0.185
*Epinephelus ongus*	1	638.00	2	23.76
*Lutjanus carponotatus*	2	274.00	2	1.69
*Lutjanus fulvus*	1	139.00	7	0.027
*Lutjanus russellii*	2	279.60	5	1.595
*Plectropomus leopardus*	2	1509.00	2	26.77
*Seriola rivoliana*	1	843.00	4	1.775
	Summary			
	*N* prey items	88	Min. mass (g)	117
	*N* individuals	33	Max. mass (g)	647

**TABLE A2:** Prey length (mm) to mass (g) conversion factors and references. Where specific prey‐type length‐weight conversion factors were not available, those closely matching the prey type were selected. All conversions are standard length (or longest body axis, where standard lengths could not be applied) to wet mass conversions unless otherwise specified. Where conversions are length to dry weight, a dry to wet weight conversion factor was applied. Equations corresponding to each prey‐type conversion factors also shown

Prey classification	a	b	Reference
Crustacean: copepod^a^, ^b^	−2.021	2.486	Kwong et al. (2018); copepods
Crustacean: megalopa^a^, ^b^	−4.838	2.651	Kwong et al. (2018); decapods
Crustacean: ostracod^a^, ^b^	−1.599	2.86	Kwong et al. (2018); copepods
Crustacean: zoea^a^, ^b^	−4.838	2.651	Kwong et al. (2018); decapods
Cephalopod: octopuA1	−2.711	2.672	Robinson et al. (2010); *Eledone cirrhosa*
Crustacean: amphipod^b^	−4.333	3.06	Robinson et al. (2010); *Iphimedia obese*
Crustacean: crab^b^	−3.427	2.875	Robinson et al. (2010); *Liocarcinus holsatus*
Crustacean: crab hermit^b^	−3.757	2.75	Robinson et al. (2010); *Colus jeffreysianus*
Crustacean: isopod^b^	−4.838	2.651	Robinson et al. (2010); *Astacilla longicornis*
Crustacean: pycnogonida^b^	−4.333	3.06	Robinson et al. (2010); *Iphimedia obese*
Crustacean: shrimp^b^	−4.988	3.011	Robinson et al. (2010); *Processa canaliculata*
Crustacean: unknown small‐bodied^b^	−3.018	2.883	Robinson et al. (2010); *Nephrops norvegicus*
Echinoderm: ophiuroidea^b^	−2.711	2.337	Robinson et al. (2010); *Ophiothrix fragilis*
Echinoderm: urchin^b^	−3.246	2.846	Robinson et al. (2010); *Echinus acutus*
Fish: body^c^	0.01	3	Standard fish estimate
Fish: body small^c^	0.01	3	Standard fish estimate
Mollusc: abalone^b^	−3.757	2.75	Robinson et al. (2010); *Colus jeffreysianus*
Mollusc: cowrie^b^	−3.757	2.75	Robinson et al. (2010); *Colus jeffreysianus*
Mollusc: gastropod^b^	−3.757	2.75	Robinson et al. (2010); *Colus jeffreysianus*
Mollusc: limpit^b^	−3.757	2.75	Robinson et al. (2010); *Colus jeffreysianus*
Mollusc: polyplacophoran^b^	−4.046	3.316	Robinson et al. (2010); *Leptochiton asellus*
Mollusc: scaphopoda^b^	−3.48	2.139	Robinson et al. (2010); *Antalis entalis*
Sessile filter‐feeder: barnacle^b^	−3.896	2.834	Robinson et al. (2010); *Scalpellum scalpellum*
Sessile filter‐feeder: bivalve^b^	−3.716	2.847	Robinson et al. (2010); *Modiolus modiolus*
Sessile filter‐feeder: bivalve Mytilidae^b^	−3.716	2.847	Robinson et al. (2010); *Modiolus modiolus*

^a^ Wet‐weight to dry‐weight conversions; subsequently converted to wet‐weights using the mean amphipod conversion factor of 0.262 from Ricciardi and Bourget (1998).

^b^ Equation: log_10_(Wet weigth (g)) = a + b * log_10_(Length (mm))

^c^ Equation: log_10_(Wet weight (g)) = log10 (a * Length (cm)^b^)

## 2 Cross‐validating gut content data with stable isotope analysis

We hypothesised that larger prey size should be predicted by not only by a predator's body size, but also by that predator's trophic position. To provide a measure of cross‐validation, we compared the size of prey consumed by a fish (derived from gut content analysis) to that fish's trophic position (derived from mixing model estimates based on stable isotope analysis (SIA) of d^15^N). Using isotopic mixing models, an estimate of Trophic Position (TP) can be derived from the nitrogen stable isotope values in an animal's tissues. Of the 325 individual fish containing measurable gut contents (from 97 species, 61 genera, and 34 families), a subset of 280 individuals (from 81 species, 57 genera, and 24 families) were also analysed for nitrogen stable isotope values. Where TP and prey size data for individual fish were available, these values were regressed using linear mixed effects models. Note, that these analyses were done at a species, rather than trophic guild level, because trophic position estimates are most meaningful at this taxonomic level. In the first model, TP and site latitude were treated as fixed effects (i.e., in package syntax: log prey mass ~ trophic position * site latitude). Latitude was included to account for potential site‐related sampling bias as fish were collected from multiple sites along a latitudinal gradient of ~30° (Figure A1; latitude may also account for temperature differences amongst these sites, although this was not explicitly tested). Random effects were (syntax: (1|log_10_ predator mass) + (1|Genus/ID)). As neither site latitude nor the interaction term were significant, we ran a second model with latitude as a random effect (syntax: 1| site latitude). In both models TP was significantly and positively related to log prey mass; however, the second model possessed the greatest explanatory power (ΔAIC 12.83). In the second model TP increased significantly with prey size (slope = 0.65, *p* = .006, Marginal *R* ^2^ / Conditional *R* ^2^ = 0.020 / 0.770).

## 3 Predicting prey mass from predator mass

The best model for predicting prey size, which we then used to project onto the community size composition (RLS survey data) is shown in Table A3, Figure A4. We also ran the same model structure, but supplementing our simplified trophic guilds (*n* = 4) with the original RLS trophic guilds (*n* = 8). When compared using AIC, the simplified trophic guild model was preferred to the RLS trophic guilds (delta AIC = −2.35). Furthermore, whilst significant differences were observed between groups in the simple trophic guild model, no significant differences were found when RLS trophic guilds were applied.

**TABLE A3:** Linear mixed effects statistics for the model used to predict log_10_ prey mass (g), according to log10 predator mass and trophic guild identity. Fixed effects: **Log 10 transformed Predator mass (g)** (continuous), **Trophic guild** (categorical, four levels: Piscivore, Invertivore, Planktivore, Herbivore) and **Site latitude** (where individual specimen ‘ID’ was collected; degrees) (continuous). All models included the nested random effects: ‘Genus/ID’, were weighted by the relative mass of prey to total gut content mass, and applied Restricted Maximum Likelihood (REML). Models built using the function ‘lmer’ in the package ‘lme4’ (Bates et al., 2012) in the statistical language R (R Development Core Team, 2021). Model syntax in package: lmer(Log10 Prey mass ~ Log10 Predator mass * Trophic guild + (1|Genus/ID), data = dat_lme, REML = T, weights = wt)

Fixed effects		Log10 prey mass (g)		
Predictors		Estimates	SE	*p*
Herbivore (Intercept)		−2.48	−3.73 – −1.23	**<.001**
Log10 Predator Mass (g)		0.11	−0.38 – 0.59	.664
Planktivore		−1.17	−2.64 – 0.29	.117
Invertivore		−1.19	−2.73 – 0.35	.131
Piscivore		−4.01	−6.67 – −1.36	.**003**
Log10 predator mass (g) * Planktivore		0.47	−0.16 – 1.09	.141
Log10 predator mass (g) * Invertivore		0.68	0.09 – 1.27	.**025**
Log10 predator mass (g) * Piscivore		2.22	1.26 – 3.18	**<.001**
Random effects				
σ^2^	0.00			
τ_00 ID:Genus_	0.62			
τ_00 Genus_	0.07			
ICC	0.99			
*N*_ID_	325			
*N*_Genus_	61			
Observations		8128		
Marginal *R* ^2^ / Conditional *R* ^2^		0.333 / 0.995 (Note: individual was included as a random effect)		

Note

Significant values at the *p* < .05 mark are often indicated in bold.

**TABLE A4:** Fixed effects structures of Linear Mixed Effects (LME) models compared using Akaike information criterion (**AIC**) and Log Likelihood (**LL**). Difference in AIC values (**ΔAIC**) from the optimal model (ΔAIC = 0), **AIC weight**, and model degrees of freedom (***df***) also shown. Only the best four models are included. The response for all models was: **Log 10 transformed individual prey mass**. Fixed effects: **Log 10 transformed Predator mass (g)** (continuous), **Trophic guild** (categorical, four levels: Piscivore, Invertivore, Planktivore, Herbivore), and **Site latitude** (where individual specimen ‘ID’ was collected; degrees) (continuous). All models included the nested random effects: ‘Genus/ID’, were weighted by the relative mass of prey to total gut content mass, and applied Restricted Maximum Likelihood (REML). Models built using the function ‘lmer’ in the package ‘lme4’ (Bates et al., 2012) in the statistical language R (R Development Core Team, 2021)

Fixed effect structure	Model syntax in R package (LMER)	*df*	ΔAIC	AIC weight	LL
Log10 (Predator mass) + Trophic Guild + Log10 (Predator mass) * Trophic guild	lmer(Log10 Prey mass ~ Log10 Predator mass * Trophic guild + (1|Genus/ID), data = data_lme, REML = T, weights = wt*)*	11	**0.0**	30068.84	−15023.42
Log10 (Predator mass) * Site latitude * Trophic guild	lmer(Log10 Prey mass ~ Log10 Predator mass * Trophic guild + Site latitude + (1| Genus /ID), data = data_lme, REML = T, weights = wt)	19	49.8	30118.65	−15040.33
Log10 (Predator mass) + Site latitude + Log10 (Predator mass) * Site latitude + Log10 (Predator mass) * Trophic guild	lmer(Log10 Prey mass ~ Log10 Predator mass * Trophic guild + Log10 Predator mass * Site latitude + (1| Genus /ID), data = data_lme, REML = T, weights = wt)	13	18.2	30087.05	−15030.52

**TABLE A5:** Random effects structures of Linear Mixed Effects (LME) models compared using Akaike information criterion (**AIC**) and Log Likelihood (**LL**). Difference in AIC values (**ΔAIC**) from the optimal model (ΔAIC = 0) and model degrees of freedom (***df***) also shown. Hierarchical nesting of terms is indicated by ‘/’. ‘**ID**’ denotes the individual specimen with which prey items are linked. ‘**Family**’, ‘**Genus**’ and ‘**Species**’ are taxonomically nested terms. All random effects structures are tested on the model: log10 (Prey mass) (g) predicted using the fixed effects: log10 (Predator mass) (g) (continuous), Trophic Guild (categorical, four levels: Piscivore, Invertivore, Planktivore and Herbivore), and the interaction of the fixed effects. The model applied Restricted Maximum Likelihood (REML) and were built using the function ‘lmer’ in the package ‘lme4’ (Bates et al., 2012) in the statistical language R (R Development Core Team, 2021). The selected model for further analyses is shaded grey. In bold are: the lowest AIC, and highest log likelihood and *R* ^2^ values

Random effects structure	*df*	ΔAIC	LL	Marginal *R* ^2^ / Conditional *R* ^2^
**Genus/ID**	11	**0.00**	−15023.42	0.333 / 0.995
Family/ID	11	0.584	−15023.71	0.344 / 0.996
Species/ID	11	0.393	−15023.62	0.331 / 0.995
Family/Species/ID	12	1.174	−15023.01	0.344 / 0.996
Family/Genus/ID	12	1.821	−15023.33	0.340 / 0.996
Genus/Species/ID	12	1.455	−15023.15	0.333 / 0.995
ID	10	3.274	−15026.06	0.317 / 0.995

## 4 Sensitivity testing cPPMR

The influence of excluding the contribution of individual trophic guilds on resulting cPPMR was investigated. Only the removal of invertivores appeared to result in considerable changes to the overall trend (Figure A4), with the resulting changes in cPPMR summary statistics shown in Table A6.

**TABLE A6:** cPPMR summary statistics (log10) from sensitivity analyses, where each trophic guild was excluded Whole community data was used in subsequent analyses

Trophic guilds excluded	Min.	1st Qu.	Median	Mean	3rd Qu.	Max.
No exclusions (i.e. whole community)	21	5751	8305	8675	12,507	15,776,588
Herbivores excluded	82	5906	7934	8367	11,146	16,640,345
Invertivores excluded	12	7018	14,533	13,196	27,517	20,191,716
Piscivores excluded	20.97	5733.81	8109.26	8419.46	12,016.40	253,152.49
Planktivores excluded	21	5267	7280	7692	10,739	15,900,561

## 5 Relationship between cPPMR and size spectra slope

Models investigating the relationship between *b* and log_10_ cPPMR treating were constructed with mean Sea Surface Temperature (SST) treated as a fixed (Table 1; Figure 2) or random (Table A7) effect. In both cases there was a significant relationship between *b* and cPPMR, however the model with mean SST as a fixed effect possessed a lower AICc (Table A8). Different random effects structure of this model were compared by either nesting Site within Year (ie. In model parlance (1|Year/Site)) or by keeping both random effects separate (i.e., (1|Year) + (1|Site)). The model with site nested in year possessed the lowest AIC (ΔAIC 57.4), consequently this random effects structure was used.

**TABLE A7:** Linear mixed effects statistics for the model used to predict fish community size spectrum slope (*b*−1), according to log_10_ cPPMR. Fixed effects: **Log_10_ cPPMR** (continuous). Random effects: **site** (as multiple transects were sometimes conducted at the same site within the same year), **year** (some sites were repeatedly sampled over years) and **mean SST** (°C). Restricted Maximum Likelihood (REML) was applied. Model was built using the function ‘lmer’ in the package ‘lme4’ (Bates et al., 2012) in the statistical language R (R Development Core Team, 2021). Model syntax in package: lmer(*b* ~ cPPMR + (1|Year/Site) + (1|mean SST), REML = T)

	*b*−1		
	Estimates	CI	*p*
*Fixed effects*			
(Intercept)	−1.12	−1.24 – −1.01	<.001
Log_10_ cPPMR	0.18	0.15 – 0.21	<.001
*Random effects*			
σ^2^	0.03		
τ_00 Site:Year_	0.01		
τ_00 Mean SST_	0.03		
τ_00 Year_	0.00		
ICC	0.53		
*N*_Site_	1220		
*N*_Year_	11		
*N*_Mean SST_	443		
Observations (transects)	5401		
Marginal *R* ^2^ / Conditional *R* ^2^	0.027 / 0.538		

**TABLE A8:** Comparison of models containing predictor combinations. The most complex model was subjected to the ‘dredge’ function (MuMIn package) in R: Linear mixed effects model containing log_10_ cPPMR and mean annual Sea Surface Temperature (Mean SST; °C). Fixed effects: **Log_10_ cPPMR** (continuous) and **Mean SST** (continuous). Random effects: **site** (as multiple transects were sometimes conducted at the same site within the same year) and **year** (some sites were repeatedly sampled over years). Restricted Maximum Likelihood (REML) was applied. Model was built using the function ‘lmer’ in the package ‘lme4’ (Bates et al., 2012) in the statistical language R (R Development Core Team, 2021). Model syntax in package: lmer(b ~ cPPMR * Mean SST + (1|Year/Site), REML = T). Model considered optimal is shaded in grey

Intercept	Mean SST	Log_10_ cPPMR	Mean SST * Log_10_ cPPMR	*df*	Log Likeli‐hood	AICc	Delta AIC	Weight
1.6650	−0.1307	−0.3968	0.0274	7	561.498	−1109.0	0.00	1
0.5044	−0.0250	0.1635		6	543.252	−1074.5	34.49	0
0.2000	−0.0230			5	482.490	−955.0	154.01	0
−1.2420		0.2166		5	349.587	−689.2	419.81	0
−0.3921				4	247.606	−487.2	621.77	0

## 6 Sensitivity testing cPPMR and Size Spectra slope relationship

### 6.1 Sensitivity to extreme values

Biological data, particularly that spanning, decades, climate regimes and a range of habitat types (as does the RLS dataset) are notoriously noisy. Whilst we accounted for temporal effects (year) and spatial effects (site and temperature) where possible, there are numerous unaccounted for variables (such as season, time of day, habitat, fishing pressure, proximity to metropolitan centres) for which we did not possess the relative data. To test the sensitivity of our results to extreme values of cPPMR (ultimately derived from RLS data) we re‐ran the above model size spectrum slope as a function of log_10_ cPPMR and temperature (with site and year as random effects) on two subsets, firstly excluding data in the 1st and 99th percentiles, secondly excluding the 5th and 95th percentiles. The removal of these data did not change the direction of the predictions, and had no or negligible impact on the marginal and conditional *R* ^2^.

Predictions and confidence intervals of linear mixed effects (LME) model for *b*−1 as a function of log_10_ cPPMR and temperature (with site and year as random effects) for the data excluding the 0.01 and 0.99 quantiles; see Figure 2 for the full dataset, note direction and significance of predictions are the same. Marginal and conditional *R* ^2^ for the model were 0.21 and 0.51. Data points represent fish communities per individual RLS transect.

**TABLE A9:** Comparison of datasets test model sensitivity to excluding extreme values. Three different datasets were compared: the full dataset (no exclusions); excluding the lowest (1st percentile) and highest (99th percentile) values; and excluding the 5th and 95th percentile tails of the data. Linear mixed effects statistics for the model used to predict fish community size spectrum slope (*b*−1), according to log_10_ cPPMR and mean annual Sea Surface Temperature (Mean SST; °C). Fixed effects: cPPMR (continuous) and Mean SST (continuous). Random effects: site (as multiple transects were sometimes conducted at the same site within the same year), and year (some sites were repeatedly sampled over years). Restricted Maximum Likelihood (REML) was applied. Model was built using the function ‘lmer’ in the package ‘lme4’ (Bates et al., 2012) in the statistical language R (R Development Core Team, 2021). Model syntax in package: lmer(b ~ cPPMR * Mean SST + (1|Year/Site), REML = T). Model used in the resulting analysis is shaded in grey

Dataset	Model factors	Coef.	CI	*p*	*N*_transects_	*N*_transects_ excluded	Marginal *R* ^2^ / Conditional *R* ^2^
All data	(Intercept)	1.67	1.03 – 2.31	**<.001**	5401	0	**0.20 / 0.51**
	Log_10_ cPPMR	−0.40	−0.56 – −0.23	**<.001**			
	Mean SST	−0.13	−0.16 – −0.10	**<.001**			
	Interaction	0.03	0.02 – 0.04	**<.001**			
1st & 99th percentile cPPMR excluded	(Intercept)	1.17	0.45 – 1.89	.**001**	5292	109 (−2%)	0.21 / 0.51
	Log_10_ cPPMR	−0.28	−0.46 – −0.09	.**003**			
	Mean SST	−0.12	−0.15 – −0.08	**<.001**			
	Interaction	0.02	0.02 – 0.03	**<.001**			
5th & 95th percentile cPPMR excluded	(Intercept)	0.80	−0.06 – 1.65	.067	4, 860	541 (−10%)	0.20 / 0.51
	Log_10_ cPPMR	−0.18	−0.40 – 0.04	.104			
	Mean SST	−0.11	−0.15 – −0.07	**<.001**			
	Interaction	0.02	0.01 – 0.03	**<.001**			
