# Supplementary material for: Community size structure varies with predator–prey size relationships and temperature across Australian reefs
Source: Ecol Evol. 2022 Apr 7;12(4):e8789. doi: 10.1002/ece3.8789 (PMC8987491; doi:10.1002/ece3.8789)
Supplement: Supplementary file 1 — SupInfo [file ECE3-12-e8789-s001.docx]

**Appendix**

**Specimen collection & gut content analysis**

Fish specimens used in this study were opportunistically collected from a variety of sites along a latitudinal gradient (Fig. A1), as part of a larger study investigating stable isotope values in reef fish tissues. Although a total of 876 individual fish (from 140 species, 83 genera, and 37 families) were sampled, only 325 individuals (from 97 species, 61 genera, and 34 families) contained ‘measurable’ (see methods for definition) gut content items, with species further categorised into trophic guilds (Table A1). Sampling targeted locally common species; the average biomass recorded on RLS surveys which the species sampled account for is shown in Fig. A2. Meanwhile, prey items from these fish were measured and lengths converted to weights as per the methods (also see Fig. A3) and Table A2.


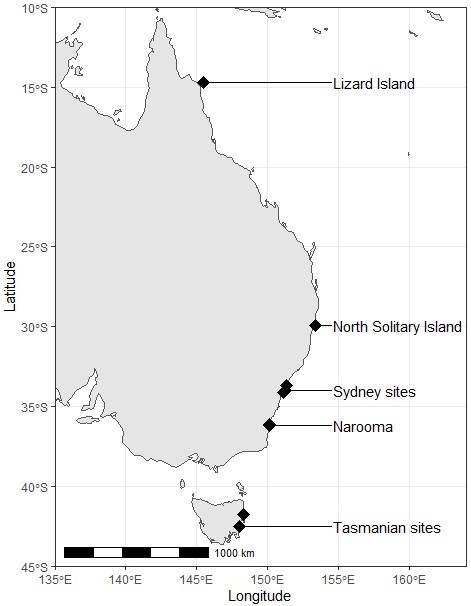


**Figure A1:** Fish collection sites, collections occurred in Spring or Autumn (2018 – 2019).

**Table A1:** Specimens collected for dietary analysis, subsequently classified within to four trophic guilds (Herbivore, Planktivore, Invertivore, Piscivore) for analysis. Note: only prey that were both identifiable and sufficiently intact to enable measurement were included in the study, and in some cases prey items were subsampled and multiplied accordingly. The number of individuals and prey items per trophic guild are summarised.

| **Species** | **N predator** | **Mean predator mass (g)** | | | | | **N prey measured per species** | **Mean prey**  **mass (g)** |
| --- | --- | --- | --- | --- | --- | --- | --- | --- |
| **Herbivore** | | | | | | | | |
| *Acanthurus dussumieri* | 4 | 795.64 | | | | | 119 | 6E-04 |
| *Acanthurus nigrofuscus* | 1 | 179.00 | | | | | 1 | 0.001 |
| *Acanthurus olivaceus* | 3 | 305.00 | | | | | 11 | 0.002 |
| *Acanthurus xanthopterus* | 4 | 1337.71 | | | | | 21 | 0.002 |
| *Aplodactylus arctidens* | 1 | 1300.00 | | | | | 4 | 0.015 |
| *Aplodactylus lophodon* | 1 | 824.00 | | | | | 1 | 0.009 |
| *Chironemus marmoratus* | 1 | 134.50 | | | | | 1 | 2E-04 |
| *Ctenochaetus striatus* | 1 | 206.00 | | | | | 2 | 4E-04 |
| *Dischistodus perspicillatus* | 3 | 138.57 | | | | | 7 | 0.006 |
| *Girella elevata* | 1 | 1287.00 | | | | | 1 | 0.006 |
| *Girella tricuspidata* | 3 | 1117.67 | | | | | 36 | 0.173 |
| *Girella zebra* | 1 | 760.00 | | | | | 3 | 5E-04 |
| *Kyphosus* spp. | 1 | 2432.00 | | | | | 1 | 0.012 |
| *Kyphosus sydneyanus* | 1 | 1000.00 | | | | | 2 | 0.076 |
| *Parma microlepis* | 11 | 157.92 | | | | | 479 | 0.004 |
| *Parma unifasciata* | 2 | 147.91 | | | | | 4 | 0.013 |
| *Prionurus maculatus* | 3 | 1306.00 | | | | | 28 | 0.003 |
| *Prionurus microlepidotus* | 4 | 196.07 | | | | | 14 | 0.016 |
| *Siganus corallinus* | 1 | 219.00 | | | | | 2 | 0.008 |
| *Siganus fuscescens* | 3 | 221.33 | | | | | 3 | 0.006 |
| *Siganus lineatus* | 5 | 585.92 | | | | | 13 | 0.006 |
| *Stegastes apicalis* | 2 | 60.50 | | | | | 2 | 0.014 |
|  | **Summary** |  | | | | |  |  |
| N prey items | | **754** | | Min. mass (g) | | | | **7** |
| N individuals | | **56** | | Max. mass (g) | | | | **2,432** |
| **Planktivore** | | | | | | | | |
| *Acanthochromis polyacanthus* | 1 | 19.00 | | | | | 9 | 6.57E-05 |
| *Atypichthys strigatus* | 11 | 24.79 | | | | | 974 | 3E-04 |
| *Caesio cuning* | 4 | 267.17 | | | | | 24 | 0.11 |
| *Heniochus* spp. | 1 | 235.50 | | | | | 3 | 0.002 |
| *Myripristis adusta* | 2 | 187.75 | | | | | 12 | 0.003 |
| *Pempheris* spp. | 3 | 67.75 | | | | | 20 | 0.05 |
| *Schuettea scalaripinnis* | 2 | 77.49 | | | | | 143 | 4.95E-05 |
| *Scorpis aequipinnis* | 4 | 462.11 | | | | | 65 | 0.017 |
| *Scorpis lineolata* | 9 | 276.05 | | | | | 57 | 0.098 |
|  | **Summary** |  | | | | |  |  |
| N prey items | | **1,312** | | | | Min. mass (g) | | **1.97** |
| N individuals | | **39** | | | | Max. mass (g) | | **69.3** |
| **Invertivore** | | | | | | | | |
| *Acanthaluteres vittiger* | 5 | 232.92 | | | | | 38 | 0.006 |
| *Acanthopagrus australis* | 5 | 395.72 | | | | | 18 | 0.153 |
| *Chaetodon flavirostris* | 2 | 130.95 | | | | | 21 | 0.005 |
| *Cheilinus fasciatus* | 3 | 399.25 | | | | | 8 | 0.025 |
| *Cheilodactylus fuscus* | 29 | 789.98 | | | | | 1676 | 0.016 |
| *Cheilodactylus spectabilis* | 10 | 1245.98 | | | | | 1864 | 0.008 |
| *Choerodon schoenleinii* | 1 | 2338.00 | | | | | 1 | 0.003 |
| *Cnidoglanis macrocephalus* | 1 | 1656.00 | | | | | 8 | 0.356 |
| *Coris gaimard* | 1 | 73.00 | | | | | 3 | 0.002 |
| *Coris picta* | 1 | 210.00 | | | | | 1 | 0.06 |
| *Dascyllus aruanus* | 2 | 4.60 | | | | | 5 | 2E-04 |
| *Diagramma labiosum* | 2 | 1027.11 | | | | | 54 | 0.021 |
| *Enoplosus armatus* | 5 | 129.54 | | | | | 324 | 0.005 |
| *Eubalichthys mosaicus* | 8 | 827.96 | | | | | 36 | 0.012 |
| *Gymnocranius* spp. | 4 | 899.82 | | | | | 34 | 0.037 |
| *Halichoeres chloropterus* | 3 | 62.18 | | | | | 11 | 0.012 |
| *Hemigymnus melapterus* | 5 | 469.07 | | | | | 42 | 0.004 |
| *Lethrinus harak* | 5 | 314.42 | | | | | 19 | 0.058 |
| *Lethrinus nebulosus* | 3 | 1567.86 | | | | | 21 | 0.049 |
| *Lethrinus* spp. | 1 | 559.00 | | | | | 2 | 0.079 |
| *Meuschenia australis* | 1 | 278.00 | | | | | 4 | 0.006 |
| *Meuschenia freycineti* | 6 | 660.55 | | | | | 198 | 0.156 |
| *Meuschenia trachylepis* | 7 | 348.81 | | | | | 367 | 0.004 |
| *Microcanthus strigatus* | 1 | 26.00 | | | | | 11 | 2E-04 |
| *Nemadactylus douglasii* | 1 | 547.00 | | | | | 268 | 0.004 |
| *Notolabrus fucicola* | 3 | 595.56 | | | | | 16 | 0.005 |
| *Notolabrus gymnogenis* | 14 | 845.89 | | | | | 389 | 0.013 |
| *Notolabrus tetricus* | 3 | 276.00 | | | | | 13 | 0.01 |
| *Ophthalmolepis lineolatus* | 9 | 218.73 | | | | | 22 | 0.046 |
| *Parupeneus barberinus* | 9 | 477.54 | | | | | 46 | 0.763 |
| *Parupeneus ciliatus* | 1 | 103.00 | | | | | 1 | 0.008 |
| *Parupeneus indicus* | 3 | 300.76 | | | | | 25 | 0.137 |
| *Parupeneus spilurus* | 8 | 648.63 | | | | | 82 | 0.077 |
| *Plectorhinchus albovittatus* | 1 | 7878.00 | | | | | 6 | 0.072 |
| *Plectorhinchus chaetodonoides* | 6 | 2114.95 | | | | | 110 | 0.018 |
| *Plectorhinchus chrysotaenia* | 1 | 1192.00 | | | | | 32 | 0.002 |
| *Plectorhinchus flavomaculatus* | 3 | 2266.76 | | | | | 37 | 0.048 |
| *Plectorhinchus gibbosus* | 2 | 4059.65 | | | | | 23 | 0.099 |
| *Plectorhinchus lineatus* | 2 | 1768.67 | | | | | 15 | 0.183 |
| *Pomacanthus sexstriatus* | 1 | 675.00 | | | | | 2 | 0.002 |
| *Pomacentrus moluccensis* | 1 | 1.97 | | | | | 3 | 2E-04 |
| *Pomacentrus* spp. | 1 | 3.34 | | | | | 6 | 0.001 |
| *Sargocentron spiniferum* | 1 | 464.00 | | | | | 1 | 2.227 |
| *Scolopsis bilineata* | 1 | 74.00 | | | | | 1 | 0.003 |
| *Scolopsis margaritifer* | 1 | 290.00 | | | | | 13 | 0.02 |
| *Scolopsis monogramma* | 6 | 395.14 | | | | | 50 | 0.01 |
| *Scorpaena jacksoniensis* | 3 | 484.33 | | | | | 3 | 0.34 |
| *Sufflamen chrysopterum* | 4 | 120.72 | | | | | 46 | 0.004 |
| *Thalassoma lutescens* | 1 | 358.00 | | | | | 1 | 0.002 |
|  | **Summary** |  | | | | |  |  |
| N prey items | | **5,974** | Min. mass (g) | | | | | **39** |
| N individuals | | **197** | Max. mass (g) | | | | | **886** |
| **Piscivore** | | | | | | | | |
| *Acanthistius ocellatus* | 8 | 855.50 | | | | | 22 | 34.59 |
| *Aulopus purpurissatus* | 1 | 1161.00 | | | | | 1 | 7.635 |
| *Aulostomus chinensis* | 1 | 430.00 | | | | | 8 | 0.093 |
| *Carangoides fulvoguttatus* | 2 | 592.40 | | | | | 10 | 1.139 |
| *Carangoides plagiotaenia* | 1 | 644.00 | | | | | 4 | 0.24 |
| *Caranx papuensis* | 2 | 1666.00 | | | | | 2 | 34.91 |
| *Cephalopholis cyanostigma* | 3 | 366.33 | | | | | 3 | 6.389 |
| *Dinolestes lewini* | 4 | 411.36 | | | | | 14 | 1.206 |
| *Epibulus insidiator* | 1 | 545.00 | | | | | 1 | 1.95 |
| *Epinephelus malabaricus* | 1 | 4139.00 | | | | | 1 | 11.19 |
| *Epinephelus merra* | 1 | 117.00 | | | | | 1 | 0.185 |
| *Epinephelus ongus* | 1 | 638.00 | | | | | 2 | 23.76 |
| *Lutjanus carponotatus* | 2 | 274.00 | | | | | 2 | 1.69 |
| *Lutjanus fulvus* | 1 | 139.00 | | | | | 7 | 0.027 |
| *Lutjanus russellii* | 2 | 279.60 | | | | | 5 | 1.595 |
| *Plectropomus leopardus* | 2 | 1509.00 | | | | | 2 | 26.77 |
| *Seriola rivoliana* | 1 | 843.00 | | | | | 4 | 1.775 |
|  | **Summary** |  | | | | |  |  |
| N prey items | | **88** | | | Min. mass (g) | | | **117** |
| N individuals | | **33** | | | Max. mass (g) | | | **647** |

**Table A2:** Prey length (mm) to mass (g) conversion factors and references. Where specific prey-type length-weight conversion factors were not available, those closely matching the prey type were selected. All conversions are standard length (or longest body axis, where standard lengths could not be applied) to wet mass conversions unless otherwise specified. Where conversions are length to dry weight, a dry to wet weight conversion factor was applied. Equations corresponding to each prey-type conversion factors also shown.

| **Prey Classification** | **a** | **b** | **Reference** |
| --- | --- | --- | --- |
| Crustacean: copepod*^1^ | -2.021 | 2.486 | Kwong et al. 2018; copepods |
| Crustacean: megalopa*^1^ | -4.838 | 2.651 | Kwong et al. 2018; decapods |
| Crustacean: ostracod*^1^ | -1.599 | 2.86 | Kwong et al. 2018; copepods |
| Crustacean: zoea*^1^ | -4.838 | 2.651 | Kwong et al. 2018; decapods |
| Cephalopod: octopuA1 | -2.711 | 2.672 | Robinson et al. 2010; *Eledone cirrhosa* |
| Crustacean: amphipod^1^ | -4.333 | 3.06 | Robinson et al. 2010; *Iphimedia obese* |
| Crustacean: crab^1^ | -3.427 | 2.875 | Robinson et al. 2010; *Liocarcinus holsatus* |
| Crustacean: crab hermit^1^ | -3.757 | 2.75 | Robinson et al. 2010; *Colus jeffreysianus* |
| Crustacean: isopod^1^ | -4.838 | 2.651 | Robinson et al. 2010; *Astacilla longicornis* |
| Crustacean: pycnogonida^1^ | -4.333 | 3.06 | Robinson et al. 2010; *Iphimedia obese* |
| Crustacean: shrimp^1^ | -4.988 | 3.011 | Robinson et al. 2010; *Processa canaliculata* |
| Crustacean: unknown small-bodied^1^ | -3.018 | 2.883 | Robinson et al. 2010; *Nephrops norvegicus* |
| Echinoderm: ophiuroidea^1^ | -2.711 | 2.337 | Robinson et al. 2010; *Ophiothrix fragilis* |
| Echinoderm: urchin^1^ | -3.246 | 2.846 | Robinson et al. 2010; *Echinus acutus* |
| Fish: body^2^ | 0.01 | 3 | Standard fish estimate |
| Fish: body small^2^ | 0.01 | 3 | Standard fish estimate |
| Mollusc: abalone^1^ | -3.757 | 2.75 | Robinson et al. 2010; *Colus jeffreysianus* |
| Mollusc: cowrie^1^ | -3.757 | 2.75 | Robinson et al. 2010; *Colus jeffreysianus* |
| Mollusc: gastropod^1^ | -3.757 | 2.75 | Robinson et al. 2010; *Colus jeffreysianus* |
| Mollusc: limpit^1^ | -3.757 | 2.75 | Robinson et al. 2010; *Colus jeffreysianus* |
| Mollusc: polyplacophoran^1^ | -4.046 | 3.316 | Robinson et al. 2010; *Leptochiton asellus* |
| Mollusc: scaphopoda^1^ | -3.48 | 2.139 | Robinson et al. 2010; *Antalis entalis* |
| Sessile filter-feeder: barnacle^1^ | -3.896 | 2.834 | Robinson et al. 2010; *Scalpellum scalpellum* |
| Sessile filter-feeder: bivalve^1^ | -3.716 | 2.847 | Robinson et al. 2010; *Modiolus modiolus* |
| Sessile filter-feeder: bivalve Mytilidae^1^ | -3.716 | 2.847 | Robinson et al. 2010; *Modiolus modiolus* |
| *Wet-weight to dry-weight conversions; subsequently converted to wet-weights using the mean amphipod conversion factor of 0.262 from Ricciardi & Bourget (1998).  ^1^Equation: log_10_(Wet weigth (g)) = a + b * log_10_(Length (mm))  ^2^Equation: log_10_(Wet weight (g)) = log10 (a * Length (cm)^b^) | | | |


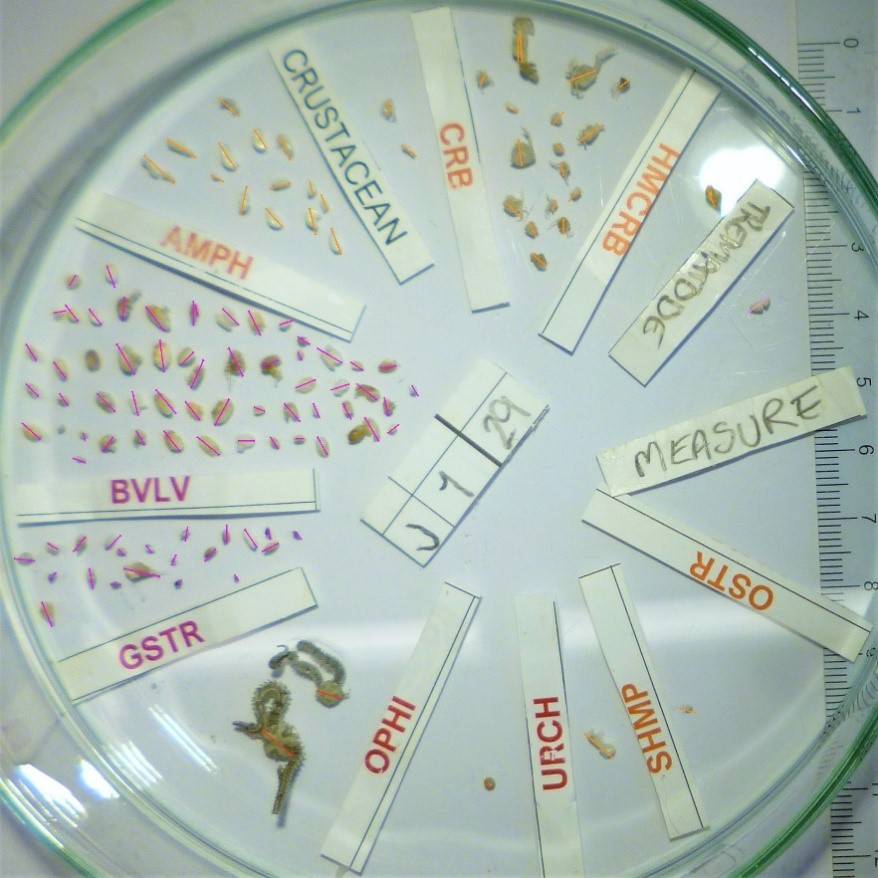


**Figure A3***:* Measurable (see methods) gut content items laid out for analysis. Items are grouped into broad categories and photographed with a scale, before being processed for measurements using the program CPCe (coloured lines indicate measurement axis). Label in centre indicates the individual fish from which items were obtained, in this case a *Cheilodactylus spectabilis* individual (fork length 350 mm, wet weight 769 g, collected from Narooma, NSW).

**Cross-validating gut content data with stable isotope analysis**

We hypothesised that larger prey size should be predicted by not only by a predator’s body size, but also by that predator’s trophic position. To provide a measure of cross-validation, we compared the size of prey consumed by a fish (derived from gut content analysis) to that fish’s trophic position (derived from mixing model estimates based on stable isotope analysis (SIA) of d^15^N). Using isotopic mixing models, an estimate of Trophic Position (TP) can be derived from the nitrogen stable isotope values in an animal’s tissues. Of the 325 individual fish containing measurable gut contents (from 97 species, 61 genera, and 34 families), a subset of 280 individuals (from 81 species, 57 genera, and 24 families) were also analysed for nitrogen stable isotope values. Where TP and prey size data for individual fish were available, these values were regressed using linear mixed effects models. Note, that these analyses were done at a species, rather than trophic guild level, because trophic position estimates are most meaningful at this taxonomic level. In the first model, TP and site latitude were treated as fixed effects (i.e., in package syntax: log prey mass ~ trophic position * site latitude). Latitude was included to account for potential site-related sampling bias as fish were collected from multiple sites along a latitudinal gradient of ~ 30^o^ (Fig. A1; latitude may also account for temperature differences amongst these sites, although this was not explicitly tested). Random effects were (syntax: (1|log_10_ predator mass) + (1|Genus/ID)). As neither site latitude nor the interaction term were significant, we ran a second model with latitude as a random effect (syntax: (1| site latitude). In both models TP was significantly and positively related to log prey mass; however, the second model possessed the greatest explanatory power (Δ AIC 12.83). In the second model TP increased significantly with prey size (slope = 0.65, P = 0.006, Marginal R2 / Conditional R2 = 0.020 / 0.770).

 
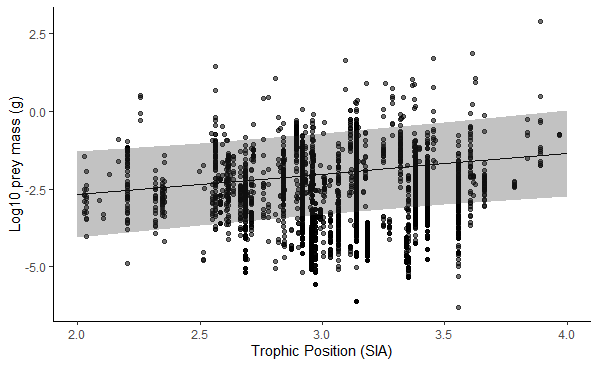


**Figure A4**: Predictions from Linear Mixed Effects model comparing individual fish Trophic Position (as calculated from nitrogen stable isotope values) and individual fish Log_10_ Prey Mass (g).

**Predicting prey mass from predator mass**

The best model for predicting prey size, which we then used to project onto the community size composition (RLS survey data) is shown in Table A3, Fig. A5. We also ran the same model structure, but supplementing our simplified trophic guilds (n = 4) with the original RLS trophic guilds (n = 8). When compared using AIC, the simplified trophic guild model was preferred to the RLS trophic guilds (delta AIC = -2.35). Furthermore, whilst significant differences were observed between groups in the simple trophic guild model, no significant differences were found when RLS trophic guilds were applied.

**Table A3.** Linear mixed effects statistics for the model used to predict log_10_ prey mass (g), according to log10 predator mass and trophic guild identity. Fixed effects: **Log 10 transformed Predator mass (g)** (continuous), **Trophic guild** (categorical, four levels: Piscivore, Invertivore, Planktivore, Herbivore) and **Site latitude** (where individual specimen ‘ID’ was collected; degrees) (continuous). All models included the nested random effects: ‘Genus/ID’, were weighted by the relative mass of prey to total gut content mass, and applied Restricted Maximum Likelihood (REML). Models built using the function ‘lmer’ in the package ‘lme4’ (Bates *et al.*, 2012) in the statistical language R (R Development Core Team, 2021). Model syntax in package: lmer(Log10 Prey mass ~ Log10 Predator mass * Trophic guild + (1|Genus/ID),  data = dat_lme, REML = T, weights=wt).

| **Fixed effects** | | **Log10 prey mass (g)** | | |
| --- | --- | --- | --- | --- |
| *Predictors* | | *Estimates* | *SE* | *p* |
| Herbivore (Intercept) | | -2.48 | -3.73 – -1.23 | **<0.001** |
| Log10 Predator Mass (g) | | 0.11 | -0.38 – 0.59 | 0.664 |
| Planktivore | | -1.17 | -2.64 – 0.29 | 0.117 |
| Invertivore | | -1.19 | -2.73 – 0.35 | 0.131 |
| Piscivore | | -4.01 | -6.67 – -1.36 | **0.003** |
| Log10 predator mass (g) * Planktivore | | 0.47 | -0.16 – 1.09 | 0.141 |
| Log10 predator mass (g) * Invertivore | | 0.68 | 0.09 – 1.27 | **0.025** |
| Log10 predator mass (g) * Piscivore | | 2.22 | 1.26 – 3.18 | **<0.001** |
| **Random Effects** | | | | |
| σ^2^ | 0.00 |  | | |
| τ_00_ _ID:Genus_ | 0.62 |  | | |
| τ_00_ _Genus_ | 0.07 |  | | |
| ICC | 0.99 |  | | |
| N _ID_ | 325 |  | | |
| N _Genus_ | 61 |  | | |
| Observations | | 8128 | | |
| Marginal R^2^ / Conditional R^2^ | | 0.333 / 0.995 (Note: individual was included as a random effect) | | |


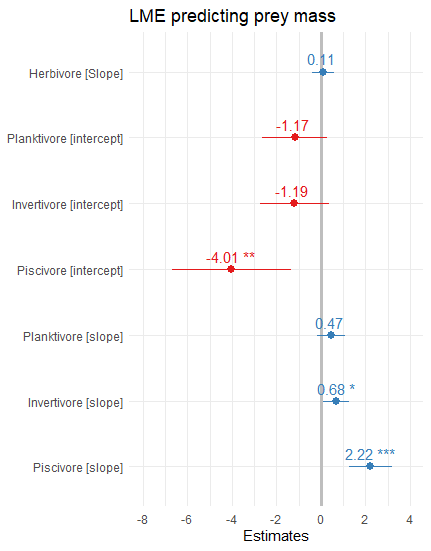


**Figure A5:** Estimates of the Linear Mixed Effects (LME) model used to predict log_10_ prey mass (g), term’s significance indicated by asterix (* <0.05; ** <0.01; *** <0.001).

**Table A4:** Fixed effects structures of Linear Mixed Effects (LME) models compared using Akaike information criterion (**AIC**) and Log Likelihood (**LL**). Difference in AIC values (**ΔAIC**) from the optimal model (ΔAIC = 0), **AIC weight**, and model degrees of freedom (**df**) also shown. Only the best four models are included. The response for all models was: **Log 10 transformed individual prey mass**. Fixed effects: **Log 10 transformed Predator mass (g)** (continuous), **Trophic guild** (categorical, four levels: Piscivore, Invertivore, Planktivore, Herbivore), and **Site latitude** (where individual specimen ‘ID’ was collected; degrees) (continuous). All models included the nested random effects: ‘Genus/ID’, were weighted by the relative mass of prey to total gut content mass, and applied Restricted Maximum Likelihood (REML). Models built using the function ‘lmer’ in the package ‘lme4’ (Bates *et al.*, 2012) in the statistical language R (R Development Core Team, 2021).

| **Fixed effect structure** | **Model syntax in R package (LMER)** | **df** | **ΔAIC** | **AIC weight** | **LL** |
| --- | --- | --- | --- | --- | --- |
| Log10 (Predator mass) **+**  Trophic Guild **+**  Log10 (Predator mass) ***** Trophic guild | lmer(  Log10 Prey mass **~**  Log10 Predator mass *****  Trophic guild **+**  (1\|Genus/ID),  data = data_lme, REML = T, weights=wt*)* | 11 | **0.0** | 30068.84 | -15023.42 |
| Log10 (Predator mass) *****  Site latitude *****  Trophic guild | lmer(  Log10 Prey mass **~**  Log10 Predator mass *****  Trophic guild **+**  Site latitude **+**  (1\| Genus /ID),  data = data_lme, REML = T, weights=wt) | 19 | 49.8 | 30118.65 | -15040.33 |
| Log10 (Predator mass) **+**  Site latitude **+**  Log10 (Predator mass) ***** Site latitude **+**  Log10 (Predator mass) ***** Trophic guild | lmer(  Log10 Prey mass **~**  Log10 Predator mass *****  Trophic guild **+**  Log10 Predator mass *****  Site latitude **+**  (1\| Genus /ID),  data = data_lme, REML = T, weights=wt) | 13 | 18.2 | 30087.05 | -15030.52 |

**Table A5:** Random effects structures of Linear Mixed Effects (LME) models compared using Akaike information criterion (**AIC**) and Log Likelihood (**LL**). Difference in AIC values (**ΔAIC**) from the optimal model (ΔAIC = 0) and model degrees of freedom (**df**) also shown. Hierarchical nesting of terms is indicated by ‘/’. ‘**ID**’ denotes the individual specimen with which prey items are linked. ‘**Family**’, ‘**Genus**’ and ‘**Species**’ are taxonomically nested terms. All random effects structures are tested on the model: log10 (Prey mass) (g) predicted using the fixed effects: log10 (Predator mass) (g) (continuous), Trophic Guild (categorical, four levels: Piscivore, Invertivore, Planktivore and Herbivore), and the interaction of the fixed effects. The model applied Restricted Maximum Likelihood (REML) and were built using the function ‘lmer’ in the package ‘lme4’ (Bates *et al.*, 2012) in the statistical language R (R Development Core Team, 2021). The selected model for further analyses is shaded grey. In bold are: the lowest AIC, and highest log likelihood and R^2^ values.

| **Random effects structure** | **df** | **ΔAIC** | **LL** | **Marginal R^2^ / Conditional R^2^** |
| --- | --- | --- | --- | --- |
| **Genus/ID** | 11 | **0.00** | -15023.42 | 0.333 / 0.995 |
| Family/ID | 11 | 0.584 | -15023.71 | 0.344 / 0.996 |
| Species/ID | 11 | 0.393 | -15023.62 | 0.331 / 0.995 |
| Family/Species/ID | 12 | 1.174 | -15023.01 | 0.344 / 0.996 |
| Family/Genus/ID | 12 | 1.821 | -15023.33 | 0.340 / 0.996 |
| Genus/Species/ID | 12 | 1.455 | -15023.15 | 0.333 / 0.995 |
| ID | 10 | 3.274 | -15026.06 | 0.317 / 0.995 |

**Sensitivity testing cPPMR**

The influence of excluding the contribution of individual trophic guilds on resulting cPPMR was investigated. Only the removal of invertivores appeared to result in considerable changes to the overall trend (Fig. A5), with the resulting changes in cPPMR summary statistics shown in Table A6.


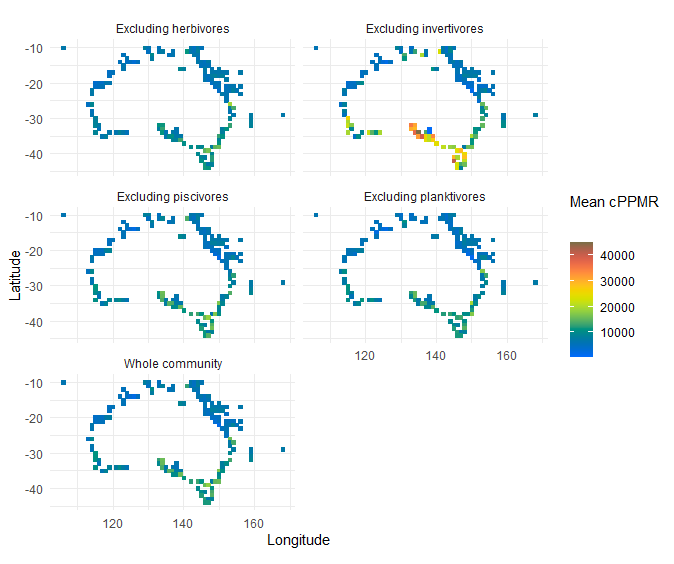


**Figure A6***:* Testing the effect of excluding individual trophic guilds (one at a time) on the resulting cPPMR. Only the exclusion of the invertivore fish guild appears to have a clearly notable impact on the cPPMR of southern sites, whilst minor changes in the cPPMR are observable in each of the other scenarios, however the overall trend remains the same. With invertivores excluded the trend observed for the ‘whole community’ is amplified rather than contradicted (i.e., the mean cPPMR in the south increases).

**Table A6:** cPPMR summary statistics (log10) from sensitivity analyses, where each trophic guild was excluded Whole community data was used in subsequent analyses.

| **Trophic guilds excluded** | **Min.** | **1^st^ Qu.** | **Median** | **Mean** | **3^rd^ Qu.** | **Max.** |
| --- | --- | --- | --- | --- | --- | --- |
| No exclusions (i.e. whole community) | 21 | 5,751 | 8,305 | 8,675 | 12,507 | 15,776,588 |
| Herbivores excluded | 82 | 5,906 | 7,934 | 8,367 | 11,146 | 16,640,345 |
| Invertivores excluded | 12 | 7,018 | 14,533 | 13,196 | 27,517 | 20,191,716 |
| Piscivores excluded | 20.97 | 5,733.81 | 8,109.26 | 8,419.46 | 12,016.40 | 253,152.49 |
| Planktivores excluded | 21 | 5,267 | 7,280 | 7,692 | 10,739 | 15,900,561 |

**Relationship between cPPMR and size spectra slope**

Models investigating the relationship between *b* and log_10_ cPPMR treating were constructed with mean Sea Surface Temperature (SST) treated as a fixed (Table 1; Fig. 2) or random (Table A7) effect. In both cases there was a significant relationship between *b* and cPPMR, however the model with mean SST as a fixed effect possessed a lower AICc (Table A8). Different random effects structure of this model were compared by either nesting Site within Year (ie. In model parlance (1|Year/Site)) or by keeping both random effects separate (i.e., (1|Year) + (1|Site)). The model with site nested in year possessed the lowest AIC (ΔAIC 57.4), consequently this random effects structure was used.

**Table A7:** Linear mixed effects statistics for the model used to predict fish community size spectrum slope (***b-1***), according to log_10_ cPPMR. Fixed effects: **Log_10_** **cPPMR** (continuous). Random effects: **site** (as multiple transects were sometimes conducted at the same site within the same year), **year** (some sites were repeatedly sampled over years) and **mean SST** (^o^ Celsius). Restricted Maximum Likelihood (REML) was applied. Model was built using the function ‘lmer’ in the package ‘lme4’ (Bates *et al.*, 2012) in the statistical language R (R Development Core Team, 2021). Model syntax in package: lmer(*b* ~ cPPMR + (1|Year/Site) + (1|mean SST),  REML = T).

|  | ***b-1*** | | |
| --- | --- | --- | --- |
| **Fixed Effects** | **Estimates** | **CI** | **p** |
| (Intercept) | -1.12 | -1.24 – -1.01 | **<0.001** |
| Log_10_ cPPMR | 0.18 | 0.15 – 0.21 | **<0.001** |
| **Random Effects** | | | |
| σ^2^ | 0.03 | | |
| τ_00_ _Site:Year_ | 0.01 | | |
| τ_00_ _Mean SST_ | 0.03 | | |
| τ_00_ _Year_ | 0.00 | | |
| ICC | 0.53 | | |
| N _Site_ | 1220 | | |
| N _Year_ | 11 | | |
| N _Mean SST_ | 443 | | |
| Observations (transects) | 5,401 | | |
| Marginal R^2^ / Conditional R^2^ | 0.027 / 0.538 | | |

**Table A8** **Comparison of models containing predictor combinations.** The most complex model was subjected to the ‘dredge’ function (MuMIn package) in R: Linear mixed effects model containing log_10_ cPPMR and mean annual Sea Surface Temperature (Mean SST; ^o^ Celsius). Fixed effects: **Log_10_** **cPPMR** (continuous) and **Mean SST** (continuous). Random effects: **site** (as multiple transects were sometimes conducted at the same site within the same year) and **year** (some sites were repeatedly sampled over years). Restricted Maximum Likelihood (REML) was applied. Model was built using the function ‘lmer’ in the package ‘lme4’ (Bates et al., 2012) in the statistical language R (R Development Core Team, 2021). Model syntax in package: lmer(b ~ cPPMR * Mean SST + (1|Year/Site),  REML = T). Model considered optimal is shaded in grey.

| **Intercept** | **Mean SST** | **Log_10_ cPPMR** | **Mean SST * Log_10_ cPPMR** | **df** | **Log Likeli-hood** | **AICc** | **Delta**  **AIC** | **Weight** |
| --- | --- | --- | --- | --- | --- | --- | --- | --- |
| 1.6650 | -0.1307 | -0.3968 | 0.0274 | 7 | 561.498 | -1109.0 | 0.00 | 1 |
| 0.5044 | -0.0250 | 0.1635 |  | 6 | 543.252 | -1074.5 | 34.49 | 0 |
| 0.2000 | -0.0230 |  |  | 5 | 482.490 | -955.0 | 154.01 | 0 |
| -1.2420 |  | 0.2166 |  | 5 | 349.587 | -689.2 | 419.81 | 0 |
| -0.3921 |  |  |  | 4 | 247.606 | -487.2 | 621.77 | 0 |

**Sensitivity testing cPPMR and Size Spectra slope relationship**

*Sensitivity to extreme values*

Biological data, particularly that spanning, decades, climate regimes and a range of habitat types (as does the RLS dataset) are notoriously noisy. Whilst we accounted for temporal effects (year) and spatial effects (site and temperature) where possible, there are numerous unaccounted for variables (such as season, time of day, habitat, fishing pressure, proximity to metropolitan centres) for which we did not possess the relative data. To test the sensitivity of our results to extreme values of cPPMR (ultimately derived from RLS data) we re-ran the above model (size spectrum slope as a function of log_10_ cPPMR and temperature (with site and year as random effects) on two subsets, firstly excluding data in the 1^st^ and 99^th^ percentiles, secondly excluding the 5^th^ and 95^th^ percentiles. The removal of these data did not change the direction of the predictions, and had no or negligible impact on the marginal and conditional R^2^.


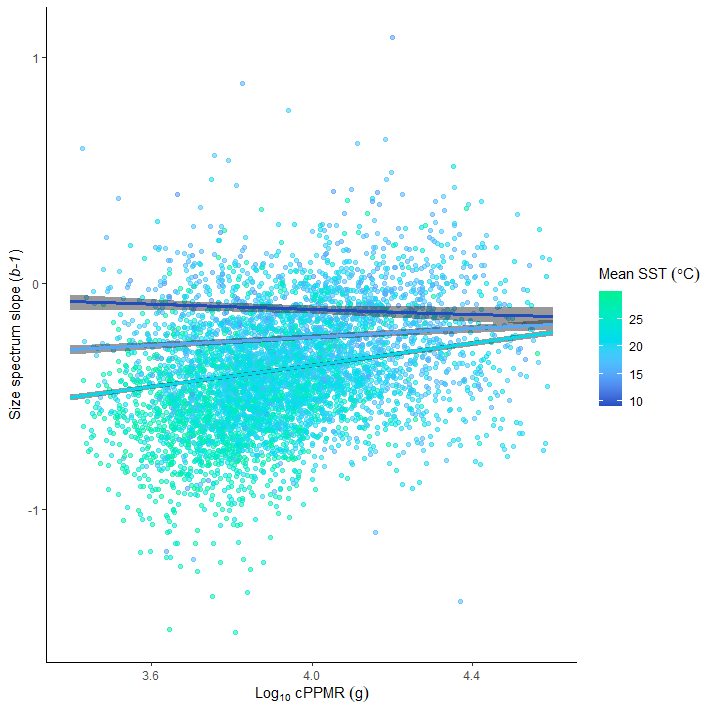


**Figure A7 Relationship between size spectrum slope (*b-1*) and log_10_ cPPMR values for fish communities, excluding 1^ST^ and 99^th^ percentiles of cPPMR.**

Predictions and confidence intervals of linear mixed effects (LME) model for *b-1* as a function of log_10_ cPPMR and temperature (with site and year as random effects) for the data excluding the 0.01 and 0.99 quantiles; see Fig. 2 for the full dataset, note direction and significance of predictions are the same). Marginal and conditional R^2^ for the model were 0.21 and 0.51. Data points represent fish communities per individual RLS transect.

**Table A9 Comparison of datasets test model sensitivity to excluding extreme values.** Three different datasets were compared: the full dataset (no exclusions); excluding the lowest (1^st^ percentile) and highest (99^th^ percentile) values; and excluding the 5^th^ and 95^th^ percentile tails of the data.  Linear mixed effects statistics for the model used to predict fish community size spectrum slope (***b-1***), according to log_10_ cPPMR and mean annual Sea Surface Temperature (Mean SST; ^o^ Celsius). Fixed effects: **cPPMR** (continuous) and **Mean SST** (continuous). Random effects: **site** (as multiple transects were sometimes conducted at the same site within the same year), and **year** (some sites were repeatedly sampled over years). Restricted Maximum Likelihood (REML) was applied. Model was built using the function ‘lmer’ in the package ‘lme4’ (Bates et al., 2012) in the statistical language R (R Development Core Team, 2021). Model syntax in package: lmer(b ~ cPPMR * Mean SST + (1|Year/Site),  REML = T). Model used in the resulting analysis is shaded in grey.

| **Dataset** | **Model factors** | **Coef.** | **C.I.** | ***p*** | **N_transects_** | **N_transects_ excluded** | **Marginal R^2^ / Conditional R^2^** | |
| --- | --- | --- | --- | --- | --- | --- | --- | --- |
| All data | (Intercept) | 1.67 | 1.03 – 2.31 | **<0.001** | 5,401 | 0 | **0.20 / 0.51** | |
|  | Log_10_ cPPMR | -0.40 | -0.56 – -0.23 | **<0.001** |  |  |  |  |
|  | Mean SST | -0.13 | -0.16 – -0.10 | **<0.001** |  |  |  |  |
|  | Interaction | 0.03 | 0.02 – 0.04 | **<0.001** |  |  |  |  |
| 1^st^ & 99^th^ percentile cPPMR excluded | (Intercept) | 1.17 | 0.45 – 1.89 | **0.001** | 5, 292 | 109  (- 2%) | 0.21 / 0.51 |  |
|  | Log_10_ cPPMR | -0.28 | -0.46 – -0.09 | **0.003** |  |  |  | |
|  | Mean SST | -0.12 | -0.15 – -0.08 | **<0.001** |  |  |  | |
|  | Interaction | 0.02 | 0.02 – 0.03 | **<0.001** |  |  |  | |
| 5^th^  & 95^th^ percentile cPPMR excluded | (Intercept) | 0.80 | -0.06 – 1.65 | 0.067 | 4, 860 | 541  (- 10%) | 0.20 / 0.51 |  |
|  | Log_10_ cPPMR | -0.18 | -0.40 – 0.04 | 0.104 |  |  |  | |
|  | Mean SST | -0.11 | -0.15 – -0.07 | **<0.001** |  |  |  | |
|  | Interaction | 0.02 | 0.01 – 0.03 | **<0.001** |  |  |  | |
